# Targeting the tumour microenvironment with an enzyme-responsive drug delivery system for the efficient therapy of breast and pancreatic cancers†
†Electronic supplementary information (ESI) available. See DOI: 10.1039/c7sc00472a



**DOI:** 10.1039/c7sc00472a

**Published:** 2017-03-08

**Authors:** Brigitte Renoux, Florian Raes, Thibaut Legigan, Elodie Péraudeau, Balkis Eddhif, Pauline Poinot, Isabelle Tranoy-Opalinski, Jérôme Alsarraf, Oleksandr Koniev, Sergii Kolodych, Stéphanie Lerondel, Alain Le Pape, Jonathan Clarhaut, Sébastien Papot

**Affiliations:** a Institut de Chimie des Milieux et des Matériaux de Poitiers (IC2MP) , Université de Poitiers , CNRS , Groupe “Systèmes Moléculaires Programmés” , 4 rue Michel Brunet, TSA 51106 , F-86073 Poitiers , France . Email: sebastien.papot@univ-poitiers.fr; b UPS no. 44 PHENOMIN TAAM-CIPA , CNRS , 3B rue de la Férollerie , F-45071 Orléans , France; c Université de Poitiers , CNRS , ERL 7368, 1 rue Georges Bonnet, TSA 51106 , F-86073 Poitiers , France; d CHU de Poitiers , 2 rue de la Miléterie, CS 90577 , F-86021 Poitiers , France; e Institut de Chimie des Milieux et des Matériaux de Poitiers (IC2MP) , Université de Poitiers , CNRS , Equipe Eau, Géochimie Organique, Santé (EGS), 4 rue Michel Brunet, TSA 51106 , F-86073 Poitiers , France; f Syndivia SAS , 650 Bd Gonthier d’Andernach , 67400 Illkirch , France

## Abstract

The development of novel therapeutic strategies allowing the destruction of tumour cells while sparing healthy tissues is one of the main challenges of cancer chemotherapy. Here, we report on the design and antitumour activity of a low-molecular-weight drug delivery system programmed for the selective release of the potent monomethylauristatin E in the tumour microenvironment of solid tumours. After intravenous administration, this compound binds covalently to plasmatic albumin through Michael addition, thereby enabling its passive accumulation in tumours where extracellular β-glucuronidase initiates the selective release of the drug. This targeting device produces outstanding therapeutic efficacy on orthotopic triple-negative mammary and pancreatic tumours in mice (50% and 33% of mice with the respective tumours cured), leading to impressive reduction or even disappearance of tumours without inducing side effects.

## Abstract 2

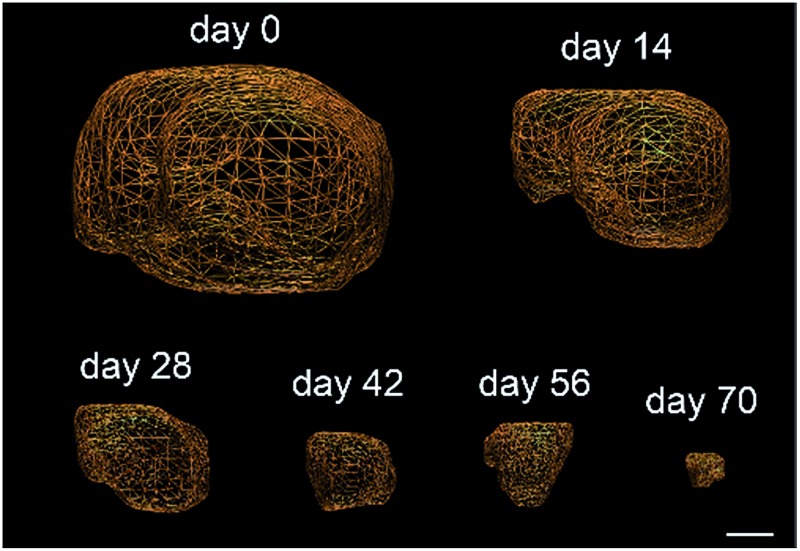
A drug delivery system targeting the tumour microenvironment produces outstanding therapeutic efficacy on triple-negative mammary and pancreatic models.

## Introduction

**Fig. 1:**
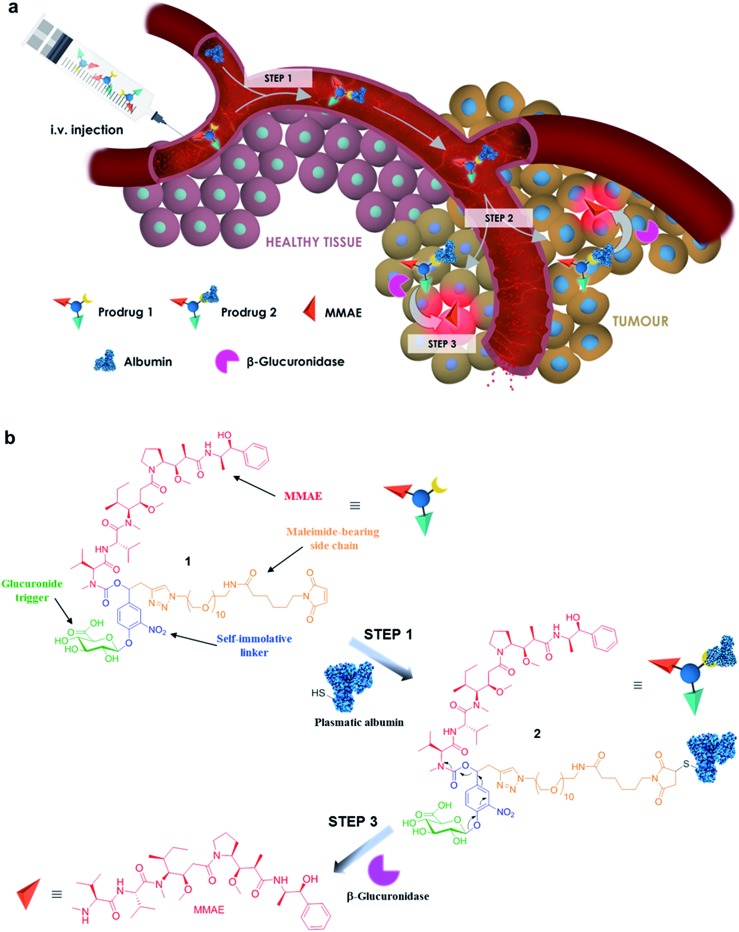
The principle of tumour targeting with the β-glucuronidase-responsive drug delivery system **1**. (a) In the blood, prodrug **1** binds to circulating albumin (step 1). The resulting macromolecule **2** accumulates passively in malignant tissues (step 2) where the cleavage of the glucuronide by extracellular β-glucuronidase triggers the release of MMAE (step 3). (b) The maleimide-bearing side chain of prodrug **1** reacts with the thiol at the cysteine 34 position of albumin through Michael addition (step 1). Hydrolysis of the glucuronide trigger by β-glucuronidase induces the release of MMAE *via* a 1,6-elimination mechanism followed by a spontaneous decarboxylation (step 3).

The controlled delivery of anticancer agents in malignant tissues is an emerging therapeutic strategy that reduces dose-limiting adverse effects associated with traditional chemotherapy. The vast majority of drug delivery systems have been designed to recognize a specific cell surface marker (*e.g.* antigens and receptors), penetrate inside cancer cells through endocytosis and trigger the release of highly toxic compounds in response to an intracellular biochemical stimulus.^1–4^ Numerous internalizing ligand- and antibody-drug conjugates have been assessed in humans, leading recently to the marketing of brentuximab vedotin^5^ and trastuzumab emtansine^6^ for applications in oncology. However, the scope of such targeting devices is restricted to only the treatment of tumours expressing a high level of the targeted cell surface marker. Here we show that the non-internalizing drug delivery system **1**, which targets the tumour microenvironment, induces remarkable anticancer activity on different animal models and is independent of the cancer cell’s surface hallmarks (Fig. 1).

The molecular assembly **1** combines a glucuronide trigger,^7,8^ a self-immolative linker,^9^ the potent monomethylauristatin E (MMAE)^10^ and a maleimide-bearing side chain in a single entity (Fig. 1a and b). Once in the blood stream, **1** binds selectively to the thiol at the cysteine 34 position of circulating albumin through Michael addition.^11,12^ The presence of albumin on the resulting drug carrier **2** prevents rapid renal elimination while ensuring passive accumulation and retention in malignant tissues due to the anatomical and pathophysiological characteristics of tumour blood vessels.^13,14^ Hydrolysis of the glycosidic bond by β-glucuronidase which selectively accumulates in the tumour microenvironment^15–21^ triggers the release of the drug *via* the self-immolative mechanism depicted in Fig. 1b. By operating in this way, the targeting system **1** mediates unprecedented anticancer activity on orthotopic triple-negative mammary and pancreatic tumours in mice, two highly lethal malignancies for which no targeted therapy is currently available.

## Results and discussion

**Scheme 1:**
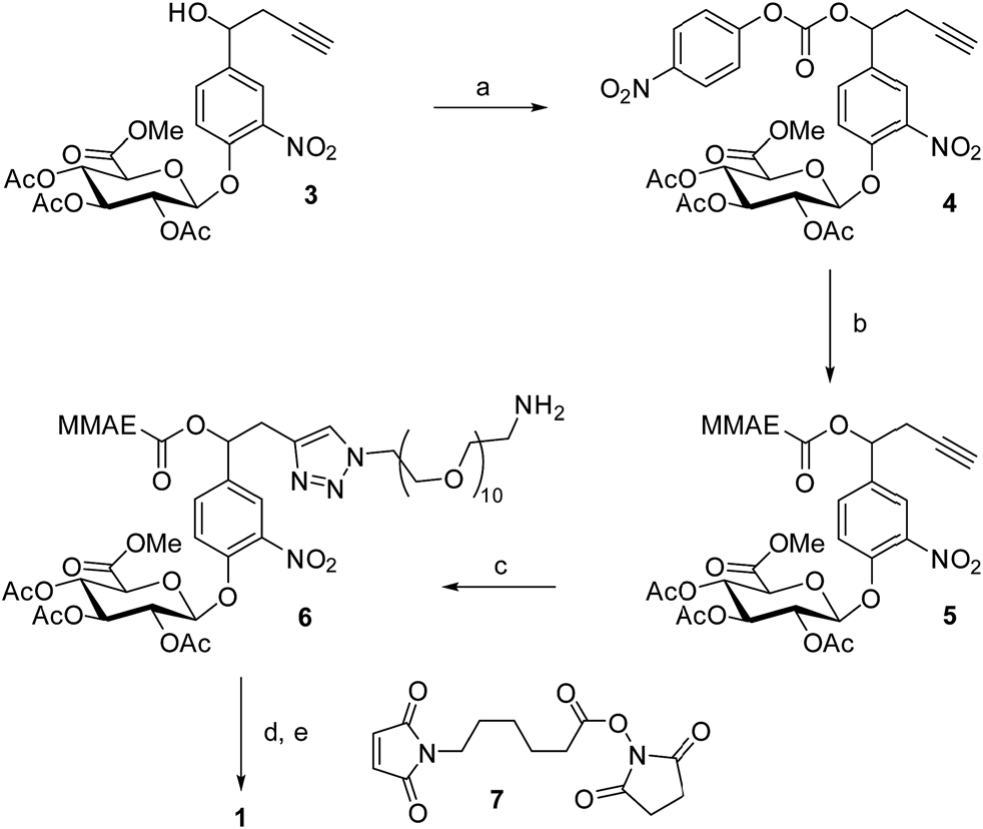
Synthesis of the glucuronide prodrug **1**. (a) 4-Nitrophenyl chloroformate, CH_2_Cl_2_, pyridine, 0 °C to rt, 1 h, quantitative; (b) MMAE, HOBt, DMF/pyridine, rt, 16 h, 94%; (c) *O*-(2-aminoethyl)-*O*′-(2-azidoethyl)nonaethylene glycol, Cu(MeCN)_4_PF_6_, CH_2_Cl_2_, rt, 20 h, 77%; (d) LiOH, H_2_O/MeOH, (e) **7**, DMSO, rt, 12 h, 33% (2 steps) after preparative-reverse phase HPLC (purity > 95%).

The drug delivery system **1** was readily accessible in only five synthetic steps from the glucuronide **3**, already described in the literature (Scheme 1).^22^ First, treatment of the benzyl alcohol **3** with 4-nitrophenyl chloroformate led to the activated carbonate **4** in quantitative yield. MMAE was then introduced *via* nucleophilic substitution in the presence of pyridine and hydroxybenzotriazole to give the carbamate **5** (94%). The latter reacted with commercially available *O*-(2-aminoethyl)-*O*′-(2-azidoethyl)-nonaethylene glycol in the presence of Cu(CH_3_CN)_4_PF_6_ to form the triazole **6** (77%). The full deprotection of the glucuronide moiety was carried out using LiOH and the crude product was engaged in the next step without purification. Finally, reaction with the hydroxysuccinimide ester **7** afforded the drug delivery system **1** with a 33% yield after purification by preparative HPLC (purity > 95%).

**Fig. 2:**
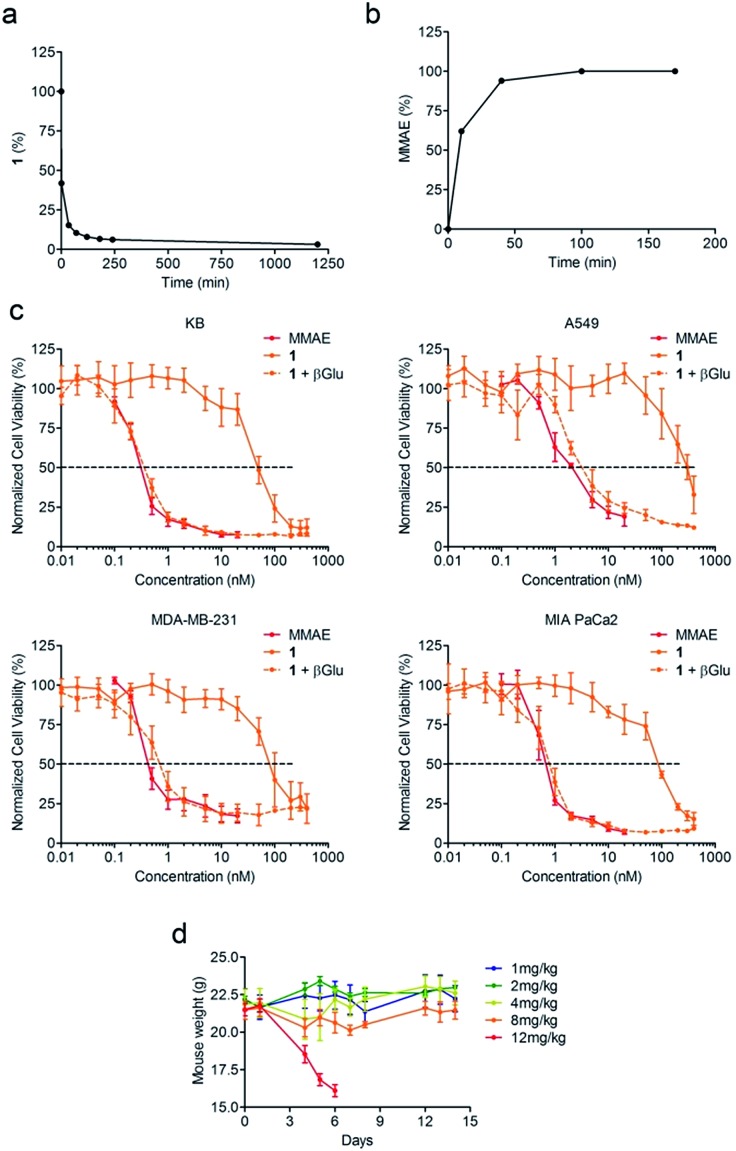
The glucuronide prodrug **1** binds covalently with HSA and efficiently releases the MMAE in the presence of β-glucuronidase. (a) Disappearance of **1** over time when placed in the presence of HSA at 37 °C. (b) Kinetics of MMAE release from **2** in the presence of β-glucuronidase (133 U mL^–1^). (c) Antiproliferative activity of MMAE and **1** with or without β-glucuronidase after 3 days of treatment. Each point shows the mean ± s.e.m. from 3 independent experiments in triplicate. (d) Mean body weights of mice treated with a single i.v. injection of **1** at 1, 2, 4, 8 or 12 mg kg^–1^ at day 0. Each point shows the mean ± s.e.m. from 3 mice.

Since enzymatic hydrolysis of the glucuronide moiety is the key step in the process of drug release, our first aim was to ascertain whether the carbohydrate trigger was still accessible to β-glucuronidase once bound to albumin. For this purpose, glucuronide **1** was incubated with human serum albumin (HSA) at 37 °C in order to form the macromolecular assembly **2**. Under these conditions, more than 90% of **1** was converted in two hours as a result of its rapid binding with the protein (Fig. 2a). Trypsin digestion followed by HPLC/HRMS analysis confirmed the formation of the coupling product **2** by the detection of the HSA peptide fragment which included the cysteine 34 linked to **1** (see the ESI†). In the presence of β-glucuronidase, prodrug **2** led to the full release of MMAE in 50 minutes, indicating that the glucuronide was a readily available substrate for the activating enzyme even with the proximity of bulky albumin (Fig. 2b). In contrast, the release of MMAE was not observed from prodrug **2** after 24 hours of incubation in the absence of β-glucuronidase.

We then examined the antiproliferative activity of glucuronide **1** against human KB, A549, MDA-MB-231 and MIA PaCa2 tumour cell lines. As a general statement, the glucuronide prodrug **1** was drastically less toxic than MMAE (Fig. 2c). On the other hand, addition of β-glucuronidase in the culture medium triggered the release of the free drug thereby restoring its initial cytotoxicity.

A tolerability study conducted in tumour free Balb/c mice demonstrated that glucuronide **1** was well tolerated up to doses of 8 mg kg^–1^ (Fig. 2d). In contrast, a 0.75 mg kg^–1^ dose of MMAE was highly toxic and induced a high rate of death in the animals, which was consistent with the previous data reported in the literature^10^ (see the ESI†). Therefore, the derivatization of MMAE in the form of prodrug **1** markedly reduced its toxicity allowing administration of at least 4-fold the lethal dose for the free drug.

**Fig. 3:**
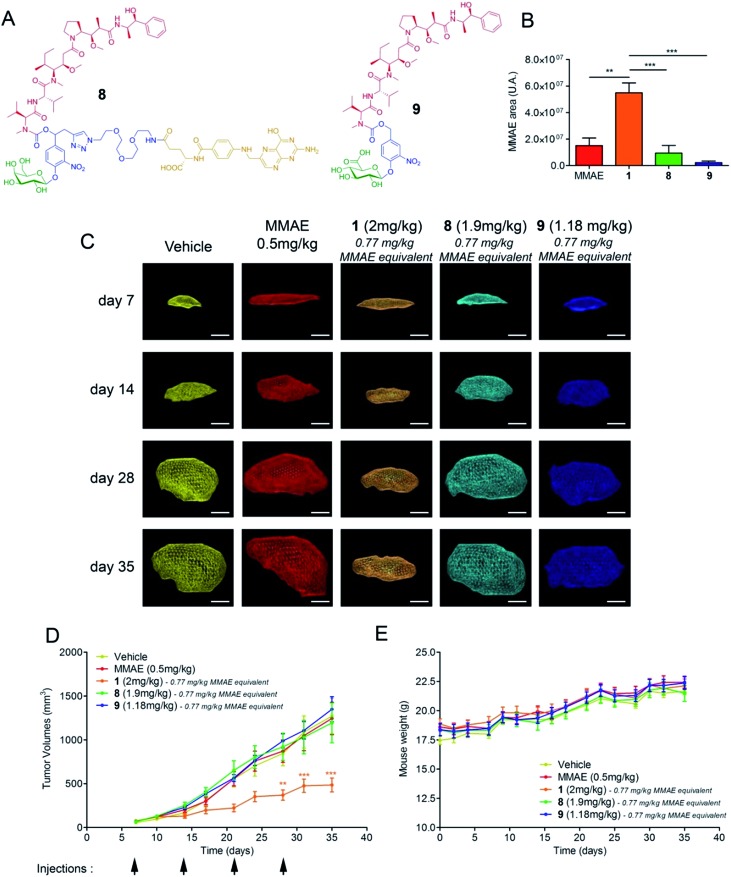
Antitumour activity of the β-glucuronidase-responsive albumin-binding prodrug **1** in mice with subcutaneous KB xenografts. (A) Structures of the β-galactosidase-responsive folate-MMAE conjugate **8** and the glucuronide prodrug of MMAE **9**. (B) Relative quantities of MMAE measured at day 21 in KB tumours of mice treated with two i.v. injections on days 7 and 14 of free MMAE (0.50 mg kg^–1^ per injection), **1**, **8** and **9** (0.77 mg kg^–1^ per injection of MMAE equivalents). Each bar shows the mean ± s.e.m. from 4 independent tumours. **P < 0.01 and ***P < 0.001; one-way analysis of variance with the Bonferroni post-test. (C) Representative volumes determined by 3D echography imaging (scale bar: 5 mm) of KB xenografts post-implantation at days 7, 14, 28 and 35 when treated with vehicle, MMAE, **1**, **8** and **9** (i.v. injection at days 7, 14, 21 and 28). (D) Tumour growth over time under therapy with vehicle, MMAE, **1**, **8** and **9**. Each point shows the mean ± s.e.m. from 8 tumour volumes. **P < 0.01 and ***P < 0.001; two-way analysis of variance with the Bonferroni post-test. (E) Mean body weights of each group of mice. Each point shows the mean ± s.e.m. from 8 mice.

We next assessed the antitumour activity of prodrug **1** in Balb/c athymic mice bearing subcutaneous KB mouth epidermal carcinoma xenografts. Indeed, as KB cells overexpress the folate receptor (FR), this tumour model is highly responsive to folate–drug conjugates.^2^ Thus, it was possible to compare the efficacy of the β-glucuronidase-catalysed drug delivery process in the tumour microenvironment with a well-established internalising approach using the drug delivery system **8** ^23^ (Fig. 3a). Additionally, we tested the glucuronide analogue **9** ^24^ that did not include a maleimide functional group with the aim of evaluating the impact of the binding to plasmatic albumin on the efficacy of tumour targeting. In the initial experiment, the animals received two doses of compounds **1**, **8** or **9** which corresponds in each case to the administration of a 0.77 mg kg^–1^ injection of MMAE on days 7 and 14 after tumour implantation. The free MMAE was also investigated, administering a 0.50 mg kg^–1^ injection, corresponding to the maximal dose before lethal toxicity. On day 21, the mice were euthanized and the relative concentration of MMAE released in the tumour from each drug delivery system was quantified. As shown in Fig. 3b, the amount of drug delivered at the tumour site was 3.6, 6 and 25-fold higher following the administration of prodrug **1** than that of MMAE, **8** and **9** respectively. In a second trial on the same animal model, each compound was injected once a week for four weeks and tumour progression was monitored by echography (Fig. 3c). The successive i.v. administrations of **1** (0.77 mg per kg per injection of MMAE equivalents) were well tolerated without any body weight loss (Fig. 3e) or detectable side effects. Furthermore, treatment with prodrug **1** produced a significant antitumour response which was notably better than that observed with the other tested molecules (Fig. 3d). In accordance with the difference observed for the β-glucuronidase-mediated deposition of MMAE in the tumour (Fig. 3b), prodrug **1** was by far more efficient than the glucuronide analogue **9**, which cannot bind covalently to circulating albumin, thus confirming the benefits brought by the linkage to the plasmatic protein. Prodrug **1** also led to superior antitumour efficacy over the folate–drug conjugate **8** while KB xenograft is the tumour model that expresses the highest level of the FR. As the targeting of FR-expressing tumours is of clinical relevance,^2^ this result suggested that the use of β-glucuronidase-responsive albumin-binding prodrugs could be an advantageous alternative to folate–drug conjugates.

As extracellular β-glucuronidase is a hallmark of a wide range of cancers in humans,^15–21^ we pursued our investigations by evaluating the therapeutic efficacy of **1** on two different orthotopic tumour models with the aim of verifying the versatility of our targeting strategy. To this end, we focused on breast and pancreatic cancers, which are two major causes of death worldwide.

**Fig. 4:**
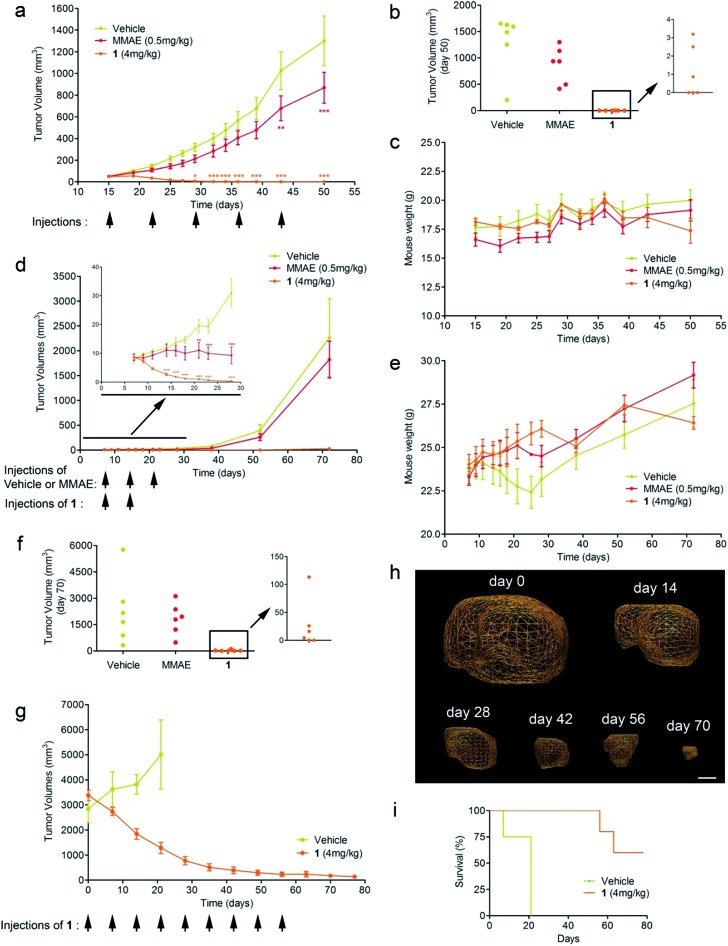
Antitumour activity of prodrug **1** on MDA-MB-231 and MIA PaCa2 orthotopic models. (a) MDA-MB-231 tumour growth inhibition under therapy with vehicle, MMAE and **1**. Each point shows the mean ± s.e.m. from 6 tumour volumes. **P < 0.01 and ***P < 0.001; two-way analysis of variance with the Bonferroni post-test. (b) Tumour volumes at day 50 of mice bearing MDA-MB-231 xenografts treated with vehicle, MMAE and **1**. (c) Mean body weights of each group of mice bearing MDA-MB-231 xenografts. Each point shows the mean ± s.e.m. from 6 mice. (d) MIA PaCa2 tumour growth inhibition under therapy with vehicle, MMAE and **1**. Each point shows the mean ± s.e.m. from 6 tumour volumes. **P < 0.01 and ***P < 0.001; two-way analysis of variance with the Bonferroni post-test. (e) Mean body weights of each group of mice bearing MIA PaCa2 xenografts. Each point shows the mean ± s.e.m. from 6 mice. (f) Tumour volumes at day 70 of mice bearing MIA PaCa2 xenografts treated with vehicle, MMAE and **1**. (g) Tumour volumes of highly hypoxic MIA PaCa2 xenografts in mice treated with vehicle and **1**. (h) Representative volumes determined by 3D echography imaging (scale bar: 5 mm) of highly hypoxic MIA PaCa2 xenografts in mice treated with **1**. Each point shows the mean ± s.e.m. from 5 tumour volumes. (i) Survival study comparing mice with 2.5–3.5 cm^3^ orthotopic MIA PaCa-2 tumours treated with prodrug **1** or untreated.

Triple-negative breast cancer (TNBC) is a clinically aggressive disease for which there is no targeted chemotherapy available. To explore the potential of prodrug **1** for the treatment of such a malignancy, human mammary MDA-MB-231 TNBC cells were orthotopically (mammary fat pad) transplanted in mice. The animals received i.v. injections of glucuronide **1** at 4 mg kg^–1^ (1.54 mg kg^–1^ equivalents of MMAE) weekly for five weeks. Following this protocol, an impressive reduction of tumour volume was observed in all treated animals (Fig. 4a). Moreover, 50% of mice treated with **1** exhibited complete remission at day 50, as assessed both by 3D echography and bioluminescence imaging (Fig. 3b), without any body weight loss or signs of toxicity (Fig. 3c). In contrast, five systemic administrations of MMAE at 0.5 mg kg^–1^ produced a moderate therapeutic effect. These data indicate that prodrug **1** is the most efficient therapeutic molecule reported to date for the treatment of TNBC in relevant preclinical models.^25,26^

We next considered prodrug **1** for the therapy of pancreatic cancer, which is the gastrointestinal malignancy with the worst prognosis and a 5 year survival rate of less than 5%. To date, chemotherapy is ineffective against this disease, prompting the need for new therapeutic approaches. Within this framework, human MIA PaCa2 cells exhibiting the four most common mutations for pancreatic adenocarcinoma^27^ were injected into the pancreas of nude mice and tumour growth was examined by 3D echography. Mice bearing intra-pancreatic tumours were treated with two 4 mg kg^–1^ doses of the glucuronide prodrug **1**. As soon as the first dose was given, tumours started to regress in all animals that received **1**, whereas treatment with MMAE (3 × 0.5 mg kg^–1^) only resulted in retardation of tumour growth (Fig. 4d and e). Tumour regression continued several days after the second administration of **1** and 33% of mice were tumour free at day 70 post-implantation, as assessed both by echography and bioluminescence imaging (Fig. 4f).

Since pancreatic cancers in humans are usually detected at a late stage and characterized by severe tumour hypoxia, rendering malignant cells resistant to chemotherapy, we conducted a new trial on mice bearing MIA PaCa2 orthotopic xenografts with sizes ranging from 2.5 to 3.5 cm^3^. As determined by photoacoustic imaging these tumours displayed strong hypoxia, therefore making this model more predictive (see the ESI†). In this experiment, mice were treated with prodrug **1** at 4 mg kg^–1^ administered weekly for nine weeks. This therapy induced remarkable antitumour activity with a dramatic reduction of the initial tumour volumes (Fig. 4g and h). Survival time also considerably increased *versus* untreated mice, for which tumour growth led rapidly to death (Fig. 3i). In comparison to molecules currently used in clinic that only delay tumour growth in the same animal model, our prodrug **1** results in an outstanding size reduction of tumours.^28,29^

Some glucuronide prodrugs have already been evaluated *in vivo* for the treatment of various solid tumours.^7,8,21,24,30–36^ However, owing to their rapid renal clearance and/or the limited potency of the targeted drug, these prodrugs have to be administered at very high doses to achieve significant anticancer activity, hampering their transfer to the clinic. Therefore, the therapeutic efficacy demonstrated by **1** represents a real breakthrough that could have significant impact in the field of drug delivery for cancer chemotherapy.

## Conclusions

Overall, this study demonstrates that the targeting of the tumour microenvironment by means of the β-glucuronidase-responsive albumin-binding prodrug **1** is a selective, efficient and potentially versatile therapeutic strategy. We believe that this approach could be employed in combination with internalizing drug delivery systems in targeted poly-chemotherapy. The efficacy observed in pre-clinical models offers a new hope for the chemotherapy of solid tumours, especially for the treatment of pancreatic ductal adenocarcinoma, for which there is an urgent need for novel therapeutic strategies.

## Author contributions

B. R. synthesised, purified and characterised the glucuronide prodrugs and carried out enzymatic reactions. F. R. designed and conducted *in vivo* studies and analysed the data. T. L. and J. A. developed early versions of the glucuronide prodrugs. I. T. O. prepared the folate–drug conjugate. B. E. undertook trypsin-digestion experiments. P. P. conducted HPLC/HRMS experiments and analysed the data. S. L. and A. L. P. designed and supervised *in vivo* experiments. O. K. and S. K. conducted pharmacokinetic studies. E. P. carried out *in vitro* biological experiments. J. C. supervised *in vitro* biological experiments and analysed the data. S. P. designed the study and wrote the manuscript.
