# Supplementary material for: Targeting the tumour microenvironment with an enzyme-responsive drug delivery system for the efficient therapy of breast and pancreatic cancers
Source: Chem Sci. 2017 Mar 8;8(5):3427–33. doi: 10.1039/c7sc00472a (PMC5417048; doi:10.1039/c7sc00472a)
Supplement: Supplementary file 1 [file SC-008-C7SC00472A-s001.pdf]

Electronic Supplementary Material (ESI) for Chemical Science.
This journal is © The Royal Society of Chemistry 2017

Supplementary Information

Targeting the Tumour Microenvironment with Enzyme-Responsive Drug Delivery System for Efficient Therapy of Breast and Pancreatic Cancers

Brigitte Renoux,[a] Florian Raes,[b] Thibaut Legigan,[a] Elodie Péraudeau,[c, d] Balkis Eddhif,[e] Pauline Poinot,[e] Isabelle Tranoy-Opalinski,[a] Jérôme Alsarraf,[a] Oleksandr Koniev,[f] Sergii Kolodych,[f] Stéphanie Lerondel,[b] Alain Le Pape,*[b] Jonathan Clarhaut,[a, d] and Sébastien Papot*[a]

[a] Institut de Chimie des Milieux et des Matériaux de Poitiers (IC2MP), Université de Poitiers, CNRS, groupe "Systèmes Moléculaires Programmés", 4 rue Michel Brunet, TSA 51106, F-86073 Poitiers, France.

[b] UPS n°44 PHENOMIN TAAM-CIPA, CNRS, 3B rue de la Férollerie, F-45071 Orléans, France.

[c] Université de Poitiers, CNRS ERL 7368, 1 rue Georges Bonnet, TSA 51106, F-86073 Poitiers, France.

[d] CHU de Poitiers, 2 rue de la Miléterie, CS 90577, F-86021 Poitiers, France.

[e] Institut de Chimie des Milieux et des Matériaux de Poitiers (IC2MP), Université de Poitiers, CNRS, Equipe Eau, Géochimie Organique, Santé (EGS), 4 rue Michel Brunet, TSA 51106, F-86073 Poitiers, France.

[f] Syndivia SAS, 650 Bd Gonthier d'Andernach, 67400 Illkirch, France. Email: enquiry@syndivia.com

Brigitte Renoux and Florian Raes contributed equally to this work.

Corresponding authors: Sébastien Papot

sebastien.papot@univ-poitiers.fr

Table of Contents

I. Chemistry Section S3

I.1.General experimental methods S3

I.2. Synthesis of prodrug 1 S4

I.3. Synthetic procedures, characterization details, ^{1}H NMR and ^{13}C NMR plots S5

II. Biology Section

II.1. Methods S14

II.2. Characterization of compound 2 S18

II.3. IC50 values of prodrug 1 S20

II.4. Tolerability studies for prodrug 1 and MMAE S20

II.5. Histopathology analysis S21

II.6. Hypoxia in the Mia PaCa2 orthotopic pancreatic tumour model S21

II.7. Pharmacokinetic study S23

I. Chemistry Section

I.1. General experimental methods

All reactions were performed under a nitrogen atmosphere. Unless otherwise stated, solvents used were of HPLC quality. Chemicals were of analytical grade from commercial sources and were used without further purification. The reaction progress was monitored on precoated silica gel TLC plates MACHEREY-NAGEL ALUGRAM® SIL G/UV$_{254}$. (0.2 mm silica gel 60). Spots were visualized under 254 nm UV light and/or by dipping the TLC plate into a solution of phosphomolybdic acid (3 g) in ethanol (100 mL) followed by heating with a heat gun. Automatic chromatographies were performed with a COMBIFLASH RF 200I TELEDYNE ISCO instrument equipped with UV detector and using flash cartridges Interchim silica 15 or 50 µm. ^{1}H and ^{13}C NMR spectra were respectively recorded at 400 MHz and 100 MHz on a Bruker 400 Avance III instrument, equipped with an ultra-shielded magnet and a BBFO 5 mm broadband probe. ^{1}H and ^{13}C NMR of compounds **6** and **1** were recorded at 500 MHz and 125 MHz on a Bruker spectrometer equipped with a cryoprobe TXI 1H-13C-15N (5mm) in the Prism platform at University of Rennes. Chemical shifts (δ) are reported in parts per million (ppm) from low to high field and referenced to residual solvent. Coupling constants (*J*) are reported in hertz (Hz). Accurate mass was determined for all derivatives through their infusion on high resolution ESI mass spectrometers in the CBM/ICOA FR2708, at the University of Orléans and in the Organic Analysis Center of IC2MP at University of Poitiers. Analytical RP-HPLC was carried out on a Dionex Ultimate 3000 system equipped with a UV/Visible variable wavelength detector and with a reverse-phase column chromatography Acclaim$^{(R)}$ (120, C18, 250x4.6 mm, 5 µm, 120 Å) at 30°C and 1mL.min^{-1}. Method **1** used a linear gradient composed of A (0.2% TFA in water) and B (CH_3CN) beginning with A/B = 80/20 v/v and reaching A/B = 0/100 v/v within 30 min. All chromatograms were recorded at 254 nm. Semi-preparative RP-HPLC was performed with a VWR LaPrep system equipped with a spectrophotometer LaPrep P314 and a preparative pump LaPrep P110. Solvent flow 4 mL.min^{-1} was applied to a semi-preparative column ACE® C18-AR (100x10 cm, 5 µm). Gradient eluent (Method **2**) was composed of A (H_2O + TFA 0,05%) and B (CH_3CN) beginning with A/B = 80/20 v/v and reaching A/B = 0/100 v/v within 30 min.)

HPLC/HRMS experiments were performed on an Accela UHPLC system coupled to a hybrid high resolution mass spectrometer Q-Exactive (Thermo Scientific). An Acclaim® C18 column (250x4.6 mm, 5 µm, 120 Å) at 30°C was used for chromatographic separation at a flow rate of 0.5 mL.min^{-1}. The column effluent was introduced into the electrospray ionisation source (ESI) of the mass spectrometer. Analyses were performed in positive ion mode. The electrospray voltage was set at 4.0 kV. The capillary and heater temperatures were 275°C and 300°C respectively. The sheath, sweep and auxiliary gas (nitrogen) flow rates were set at 35, 10 and 20 (arbitrary units). Analysis of data was performed with Xcalibur software. HPLC/HRMS/MS (Q-Exactive) experiments were performed using a linear gradient composed of A (0.1% formic acid in water) and B (0.1% formic acid in CH_3CN) (Method **3**). After injection of 20 µL of sample, compounds were separated through a linear gradient starting from 20% of B and reaching 100% of B within 30 min.

I.2. Synthesis of prodrug 1.

Prodrug **1** was prepared according to the following strategy*:

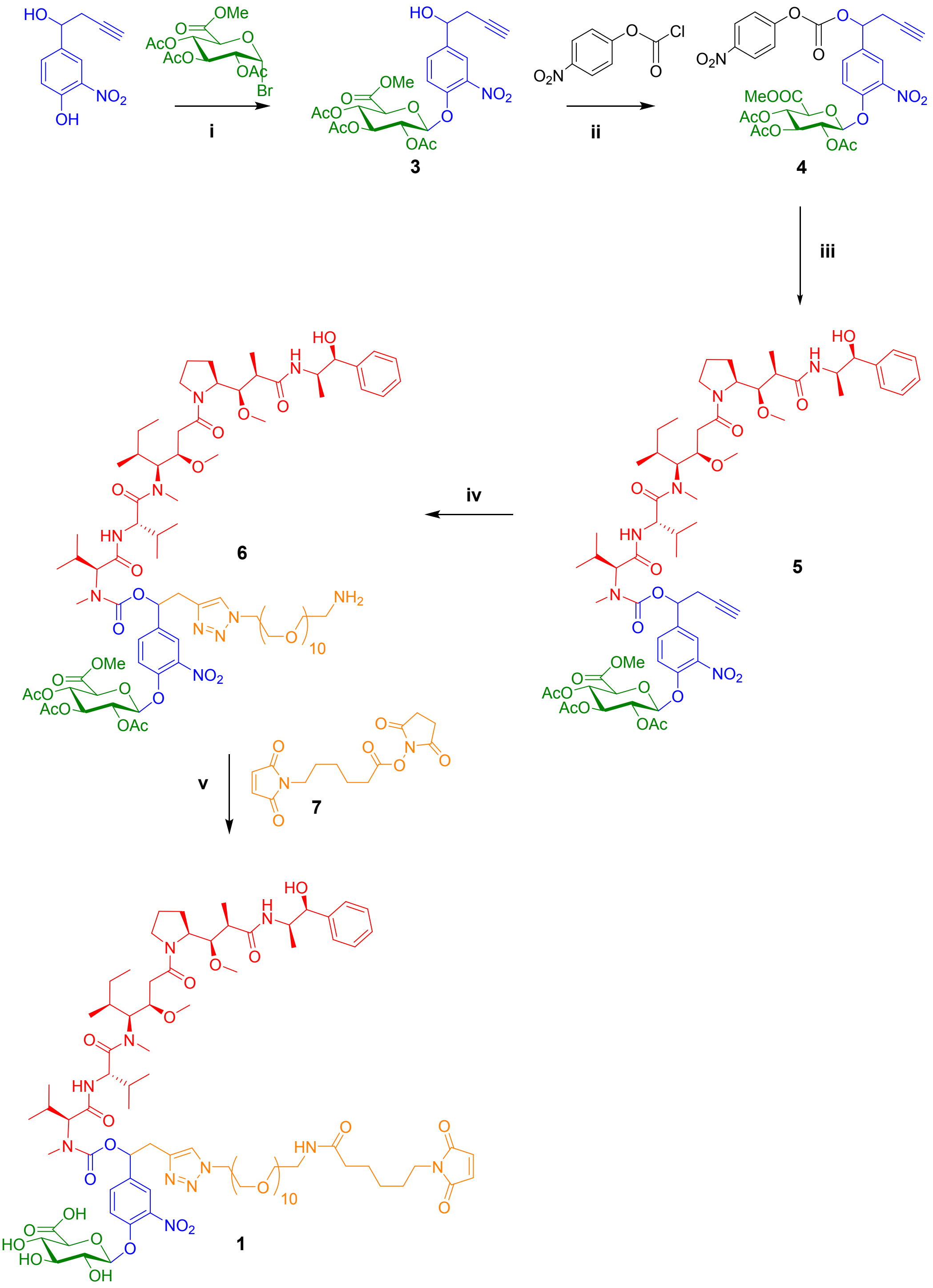

Scheme S1. *Reagents and conditions:* (i) Ag_2CO_3, HMTTA, CH_3CN, 66% (ii) 4-nitrophenyl chloroformate, CH_2Cl_2, pyridine, 0°C to rt, 1 h, quantitative; (iii) MMAE, HOBt, DMF/pyridine, rt, 16 h, 94% (iv) $Cu(MeCN)_4PF_6$, O-(2-aminoethyl)-O'-(2-azidoethyl)nonaethylene glycol, CH_2Cl_2, rt, 20 h, 77%; (v) *a)* LiOH, H_2O/MeOH, *b)* **7**, DMSO, rt, 12 h, 33% (2 steps) after preparative-reverse phase HPLC, purity > 95%.

* Compound **3** was prepared as previously described in the literature.[20]

I.3. Synthetic procedures, characterization details, ^{1}H NMR and ^{13}C NMR plots

Compound 4

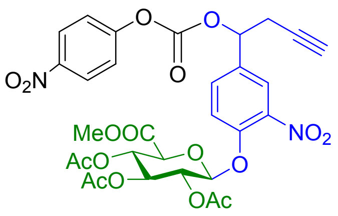

4

To a solution of **3** (180 mg, 0.34 mmol) and 4-nitrophenyl chloroformate (140 mg, 0.68 mmol) in dry dichloromethane (3.5 mL) was added pyridine (77 µL, 0.87 mmol) at 0°C. The mixture was stirred 1 hour at room temperature, hydrolyzed with saturated aqueous $NaHCO_3$. The mixture was extracted three times with dichloromethane and the combined organic layers were dried over $MgSO_4$, filtrated and concentrated in *vacuo*. Purification by column chromatography over silica gel (petroleum ether/ethyl acetate 6/4) afforded **4** (235 mg, 0.343 mmol, quantitative yield) as a mixture of two diastereoisomers (white solid).

^{1}H NMR (400 MHz, $CDCl_3$):
δ 8.3 (d, 2H, J = 9.1 Hz), 7.9 (d, 1H, J = 1.8 Hz), 7.65 (dd, 1H, J = 8.7 Hz and J = 1.8 Hz), 7.4 (m, 3H), 5.8 (t, 1H, J = 6.5 Hz), 5.3 (m, 4H), 4.2 (m, 1H), 3.7 (s, 3H), 2.90 (m, 2H), 2.10 (s, 3H), 2.09 (t, J = 2 Hz, 1H), 2.07 (s, 3H), 2.06 (s, 3H).

^{13}C NMR (100 MHz, $CDCl_3$):
δ 170.1, 169.5, 169.3, 166.8, 155.3, 149.6, 145.7, 141.1, 133.4, 132.4, 132.3, 125.5, 123.8, 121.8, 119.9, 99.4, 77.2, 72.6, 71.0, 70.2, 68.7, 53.2, 26.1, 20.4.

HRESI-MS: m/z 711.1280 (calcd. for $C_{30}H_{28}O_{17}N_2Na$ 711.1286 $[M+Na]^+$)

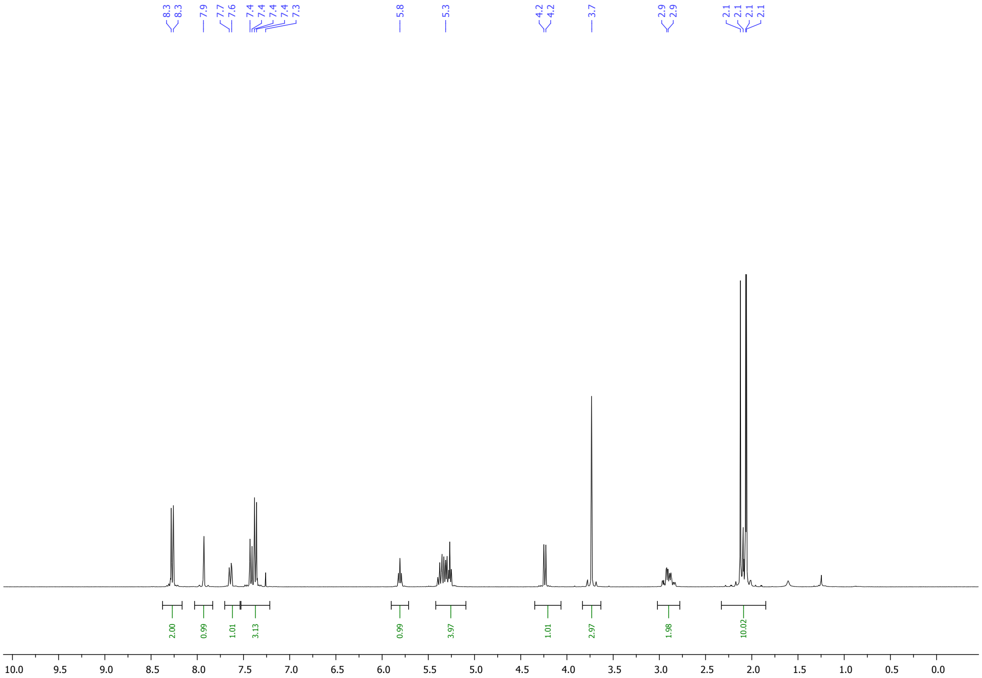

^{1}H NMR spectrum (400 MHz. 298 K. $CDCl_3$) of **4**

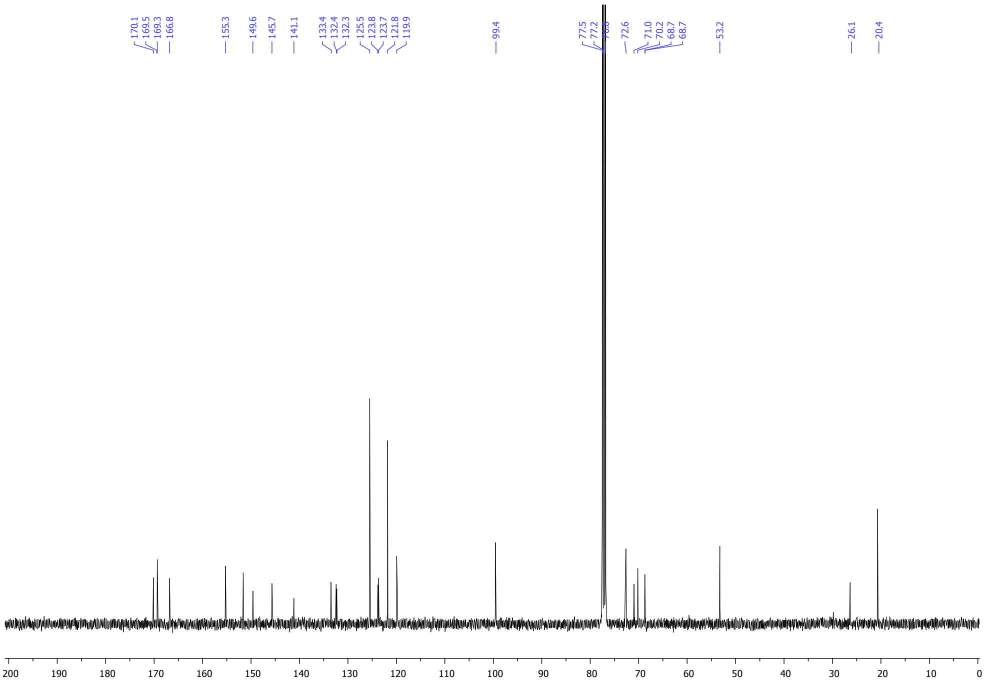

^{13}C NMR spectrum (100 MHz. 298 K. $CDCl_3$) of **4**

Compound 5

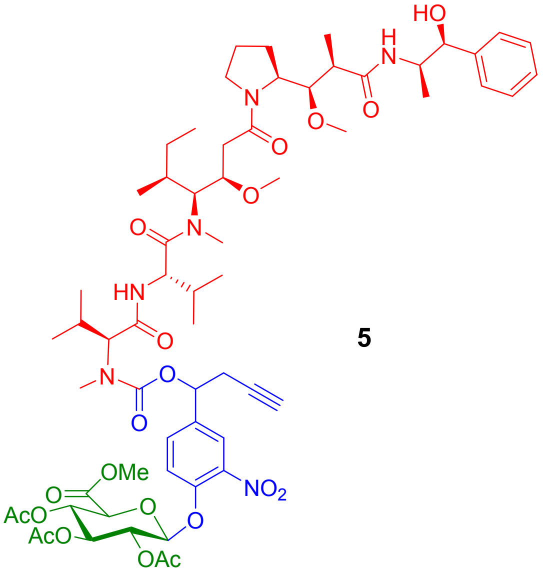

5

Carbonate **4** (57.5 mg, 0.0835 mmol) and MMAE (60 mg, 1 equiv.) were dissolved in dry DMF (1.6 mL) and pyridine (0.4 mL). HOBt (11.3 mg, 0.0835 mmol) was added. The mixture was stirred at room temperature for 16 hours. Solvents were removed under reduced pressure and crude material was purified by column chromatography over silica gel (gradient elution 2% to 5% MeOH in CH_2Cl_2) to give **5** (99 mg, 0.078 mmol, 94 %) as a white solid.

Rt = 23.43 min (Method **1**)

^{1}H NMR (400 MHz, $CDCl_3$):
δ 7.75 (m, 1H), 7.49 (m, 1H), 7.30-7.16 (m, 6H), 6.48-6.32 (m, 1H), 5.72 (m, 1H), 5.26-5.12 (m, 4H), 4.88 (s, 1H), 4.68-4.52 (m, 1H), 4.19-3.97 (m, 6H), 3.78 (m, 1H), 3.67 (s, 3H), 3.45 (m, 1H), 3.33-3.22 (m, 7H), 3.05-2.67 (m, 8H), 2.42-2.29 (m, 2H), 2.2-2.12 (m, 1H), 2.05-1.93 (m, 16H), 1.78 (m, 3H), 1.26-1.15 (m, 5H), 0.96-0.73 (m, 19H), 0.59 (m, 1H), 0.51 (m, 1H).

^{13}C NMR (100 MHz, $CDCl_3$):
δ 174.7, 173.0, 172.9, 170.7, 170.1, 169.7, 169.6, 169.4, 169.3, 166.8, 156.0, 155.9, 149.0, 141.4, 141.3, 141.1, 135.9, 135.7, 132.2, 132.0, 128.4, 128.1, 127.4, 126.4, 126.3, 123.2, 119.9, 119.8, 99.9, 99.7, 82.3, 82.1, 78.9, 78.6, 78.4, 75.9, 73.3, 73.2, 72.6, 71.9, 71.2, 70.3, 68.8, 65.6, 65.4, 65.2, 61.0, 60.3, 60.2, 58.1, 54.0, 53.9, 53.2, 51.7, 47.9, 45.0, 37.7, 33.5, 29.8, 29.7, 29.5, 26.7, 26.3, 26.2, 25.8, 25.1, 25.0, 20.7, 20.6, 19.5, 19.4, 18.6, 16.1, 14.5, 14.4, 14.0, 11.0.

HRESI-MS: *m/z* 1289.6051 (calcd. for $C_{63}H_{90}N_6O_{21}Na$ 1289,6057 $[M+Na]^+$)

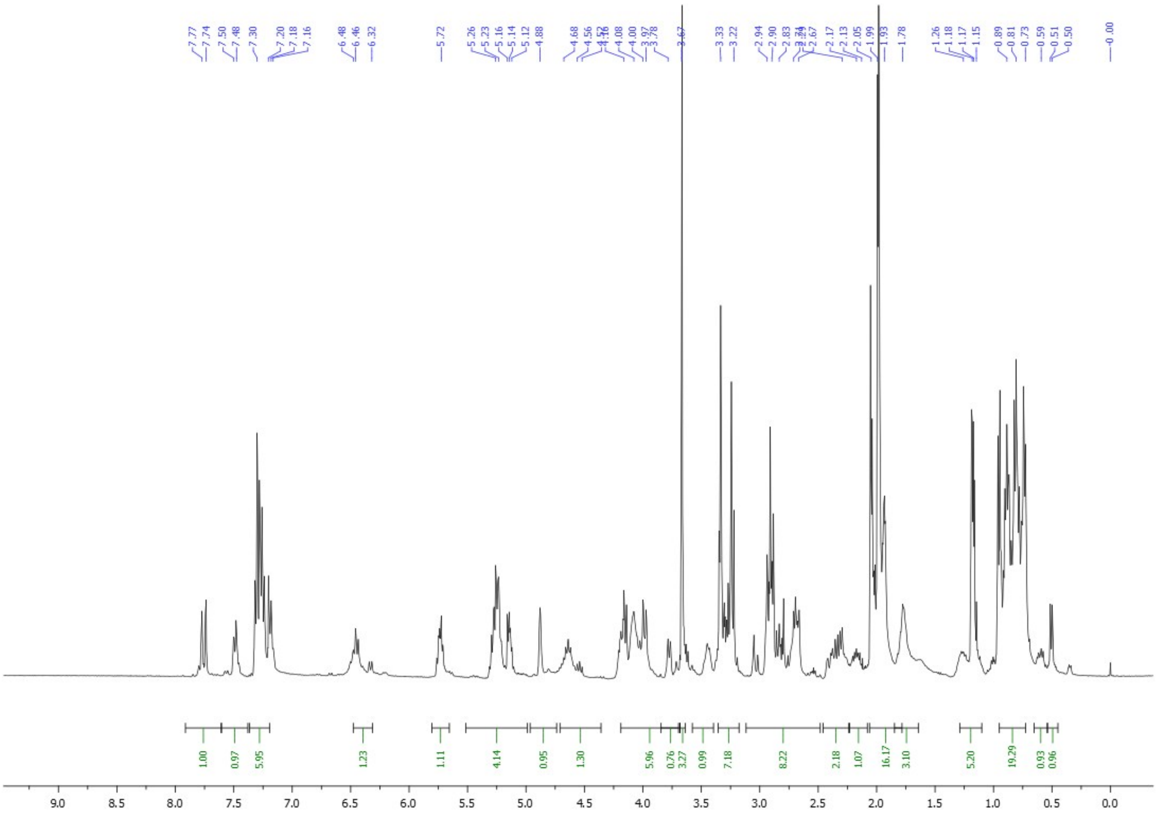

^{1}H NMR spectrum (400 MHz. 298 K. $CDCl_3$) of **5**

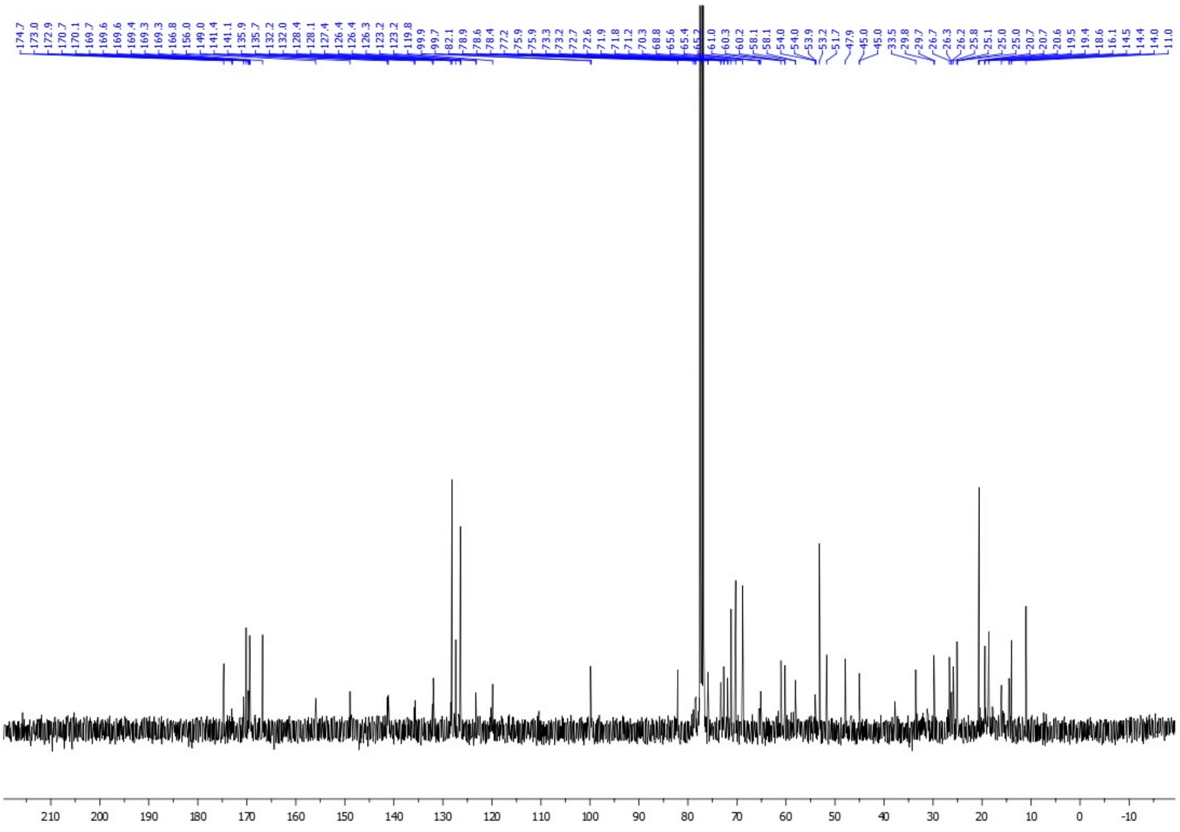

^{13}C NMR spectrum (100 MHz. 298 K. $CDCl_3$) of **5**

Compound 6

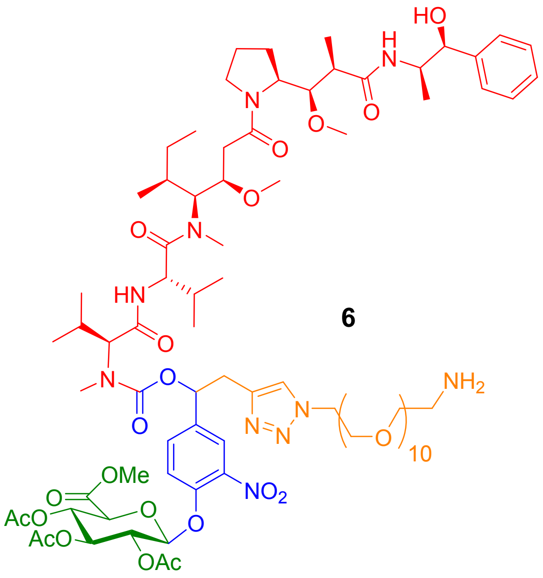

6

To a solution of **5** (70 mg, 0.055 mmol) and *O*-(2-aminoethyl)-*O*'-(2-azidoethyl)nonaethylene glycol (29.3 mg, 1.01 equiv.) in CH_2Cl_2 (2.8 mL) was added $Cu(MeCN)_4PF_6$ (30 mg, 0.08 mmol). The mixture was stirred at room temperature for 20 hours. 400 mg of QuadraPure IDA resin were then added and the suspension was stirred for 1 hour. After filtration and removal of solvent under reduced pressure, the crude product was purified by column chromatography over silica gel (gradient elution 2% to 10% MeOH in CH_2Cl_2) to give the amine **6** (75.8 mg, 0.042 mmol, 77 %).

Rt = 18.21 min (Method **1**)

^{1}H NMR (500 MHz MHz, DMSO-*d6*):
δ 8.73-8.47 (m, 0.5H), 8.19-7.55 (m, 5.5H), 7.34-7.21 (m, 5H), 5.98-5.71 (m, 2H), 5.51-5.39 (m, 1H), 5.13 (m, 1H), 4.78-4.75 (m, 1H), 4.68-4.48 (m, 5H), 4.21-3.99 (m, 2H), 3.81-3.75 (m, 2H), 3.67-3.46 (m, 48H), 3.29-3.16 (m, 11H), 3.11-2.76 (m, 7H), 2.45-2.40 (m, 1H), 2.30-2.03 (m, 10H), 1.81 (m, 3H), 1.56 (m, 2H), 1.27 (m, 7H), 1.08-0.75 (m, 20H), 0.69-0.49 (m, 2H).

^{13}C NMR (125 MHz, DMSO-*d6*):
δ 173.3, 173.2, 170.5, 170.3, 169.7, 167.8, 165.6, 156.0, 155.5, 155.4, 148.6, 148.5, 144.6, 14 3.8, 142.8, 142.5, 141, 138.2, 137.1, 133.8, 130.3, 128.7, 127.7, 127.4, 124.5, 124.2, 123.9, 12 3.5, 123.3, 118.4, 118.2, 99.0, 98.7, 98.6, 86.4, 82.5, 78.6, 77.9, 77.7, 75.7, 75.6, 74.9, 73.4, 7 2.0, 71.6, 70.7, 70.2, 69.8, 69.7, 67.6, 64.1, 64.0, 63.6, 61.9, 61.2, 61.1, 59.6, 59.1, 58.1, 57, 5 6.6, 55.9, 55.0, 53.6, 50.9, 50.7, 50.2, 50.1, 49.6, 48.2, 47.2, 44.7, 44.1, 38.0, 36.0, 32.8, 32.5, 32.3, 31.1, 30.8, 30.5, 30.2, 30.0, 29.7, 27.6, 27.4, 26.3, 25.3, 24.1, 23.1, 21.2, 21.2, 21.1, 19.8, 19.7, 19.5, 19.3, 18.9, 18.7, 16.9, 16.5, 16.4, 16.3, 16.0, 14.9, 11.3.

HRESI-MS: m/z 1793.9445 (calcd. for $C_{85}H_{137}N_{10}O_{31}$ 1793.9451 $[M+H]^+$)

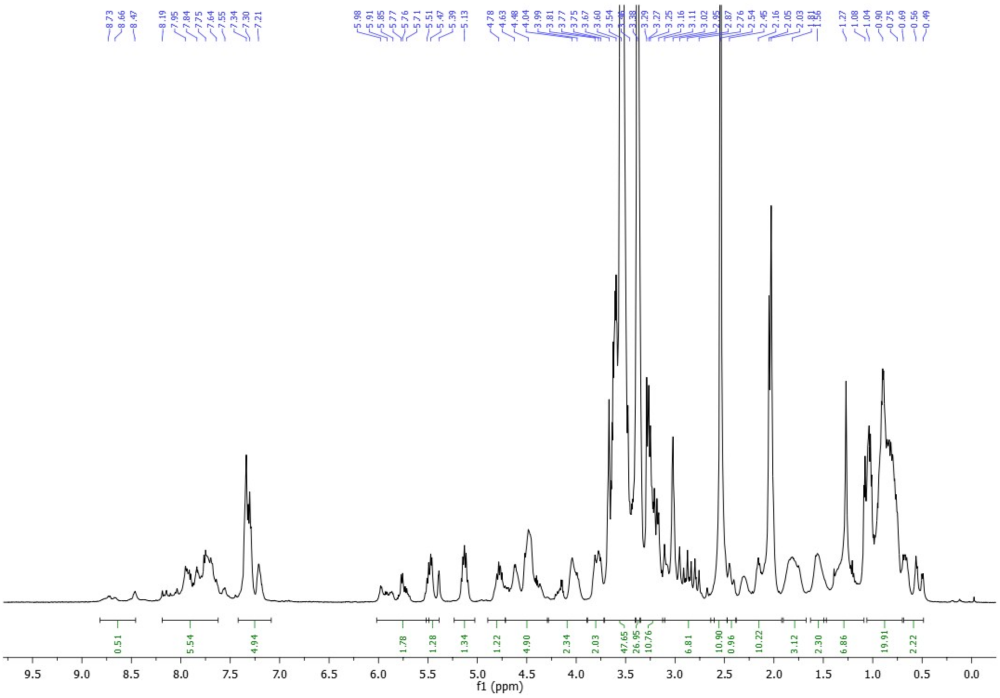

^{1}H NMR spectrum (500 MHz. 298 K. DMSO-*d6*) of **6**

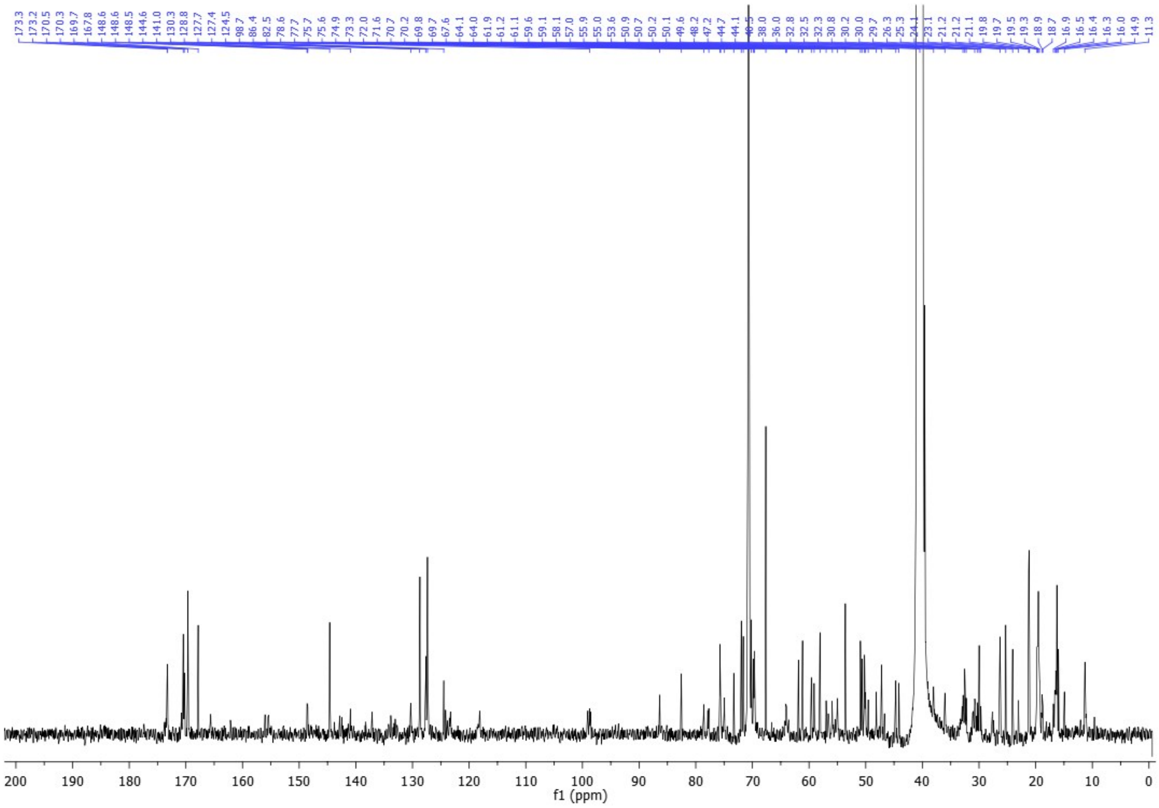

^{13}C NMR spectrum (125 MHz. 298 K. DMSO-*d6*) of **6**

Prodrug 1

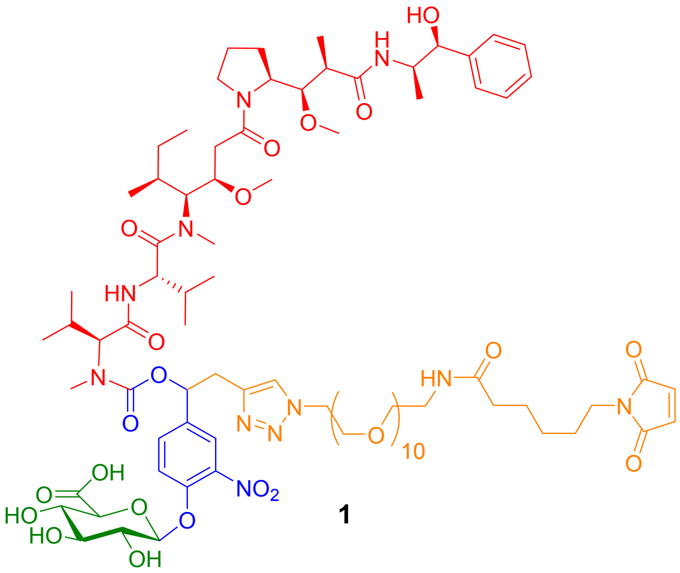

1

6 (53 mg, 0.029 mmol) was dissolved in MeOH (2.2 mL) at 0°C. A solution of lithium hydroxide monohydrate (10.6 mg, 0.259 mmol) in water (2.2 mL) was cooled at 0°C and added dropwise. The mixture was stirred until disappearance of starting material (30 min). Then hydrolysis was performed with IRC-50 acidic resin. After filtration, the solvents were removed under reduced pressure. The crude product was dissolved in DMSO (0.7 mL) and **7** (1.2 equiv., 11 mg) was added. The mixture was stirred at room temperature for 12 hours and the solvent was removed under reduced pressure. High degree of purity for **1** was obtained using preparative-reverse phase HPLC (Method **2**, 18 mg, 33%, purity > 95%).

Rt = 15.22-15.44 min (Method **1**)

^{1}H NMR (500 MHz MHz, DMSO-*d*6):
δ 8.73-8.65 (m, 0.5H), 8.50 (m, 0.5H), 8.21-8.16 (m, 0.5H) 8.12-8.04 (m, 0.5H), 7.95-7.75 (m, 3H), 7.70-7.41 (m, 2H), 7.34-7.21 (m, 6H), 7.04 (m, 2H), 5.97-5.85 (m, 1H), 5.51-5.22 (m, 4H), 4.81-4.72 (m, 1H), 4.63-4.33 (m, 5H), 4.22-3.98 (m, 4H), 3.81-3.74 (m, 3H), 3.66-3.48 (43H), 3.28-3.16 (m, 10H), 3.12-2.69 (m, 7H), 2.45 (m, 1H), 2.30 (m, 1H), 2.16-2.05 (m,4H), 1.84-1.75 (m, 3H), 1.58-1.49 (m, 7H), 1.38-1.18 (m, 3H), 1.08-0.64 (m, 26H), 0.49 (m, 1H).

^{13}C NMR (125 MHz, DMSO-*d*6):
δ 172.4, 172.3, 172.1, 171.1, 169.9, 168.8, 157.9, 157.7, 155.2, 154.6, 154.5, 148.6, 143.7, 143.0, 141.8, 140.2, 139.7, 134.5, 127.8, 126.8, 126.7, 126.4, 123.5, 123.2, 116.2, 100.1, 99.7, 85.4, 81.6, 77.7, 76.8, 75.8, 75.4, 74.8, 74.1, 72.7, 71.2, 69.8, 69.7, 69.6, 69.2, 68.9, 63.3, 63.2, 62.6, 61.0, 60.3, 58.7, 58.2, 57.2, 55.0, 54.2, 49.8, 49.3, 49.2, 47.2, 46.3, 43.8, 43.2,39.5, 38.5, 37.0, 35.0, 32.2, 31.9, 31.6, 30.1, 29.7, 29.3, 27.8, 26.8, 25.4, 24.8, 24.4, 23.1, 18.9, 18.7, 18.6, 18.5, 18.1, 18.0, 15.7, 15.5, 15.3, 15.0, 10.4.

HRESI-MS: m/z 923.9892 (calcd. for $C_{88}H_{141}N_{11}O_{31}$ 923.9897 $[M+2H]^{2+}$)

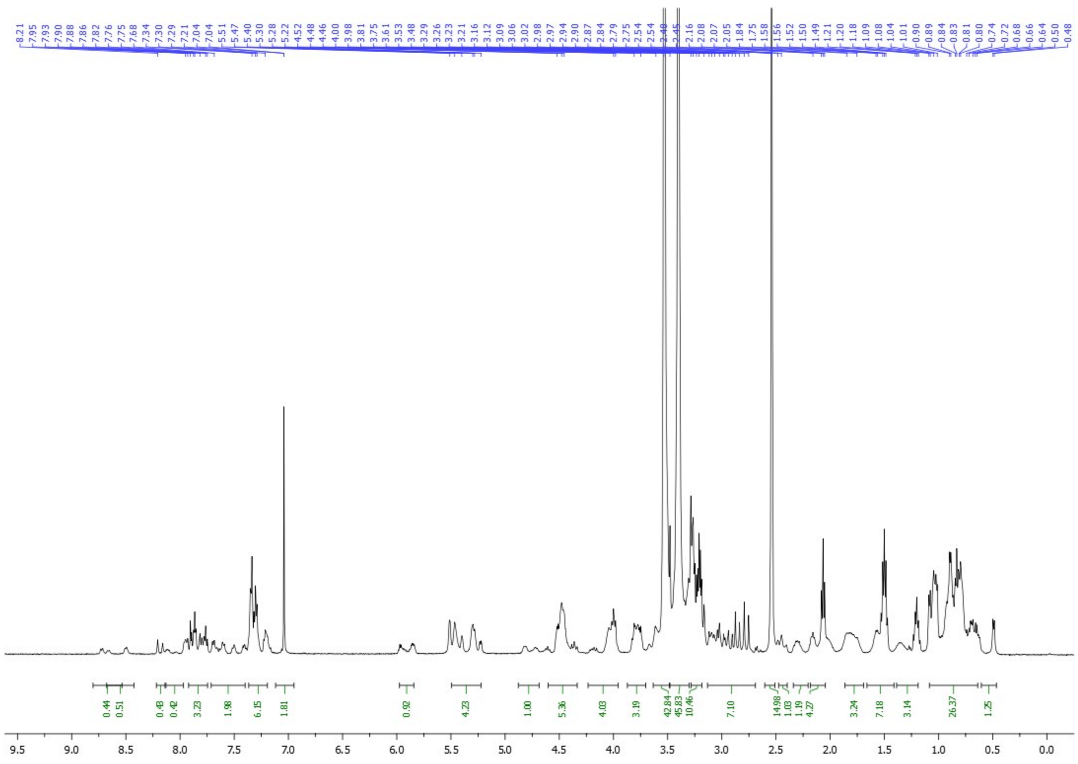

^{1}H NMR spectrum (500 MHz. 298 K. DMSO-*d*6) of **1**

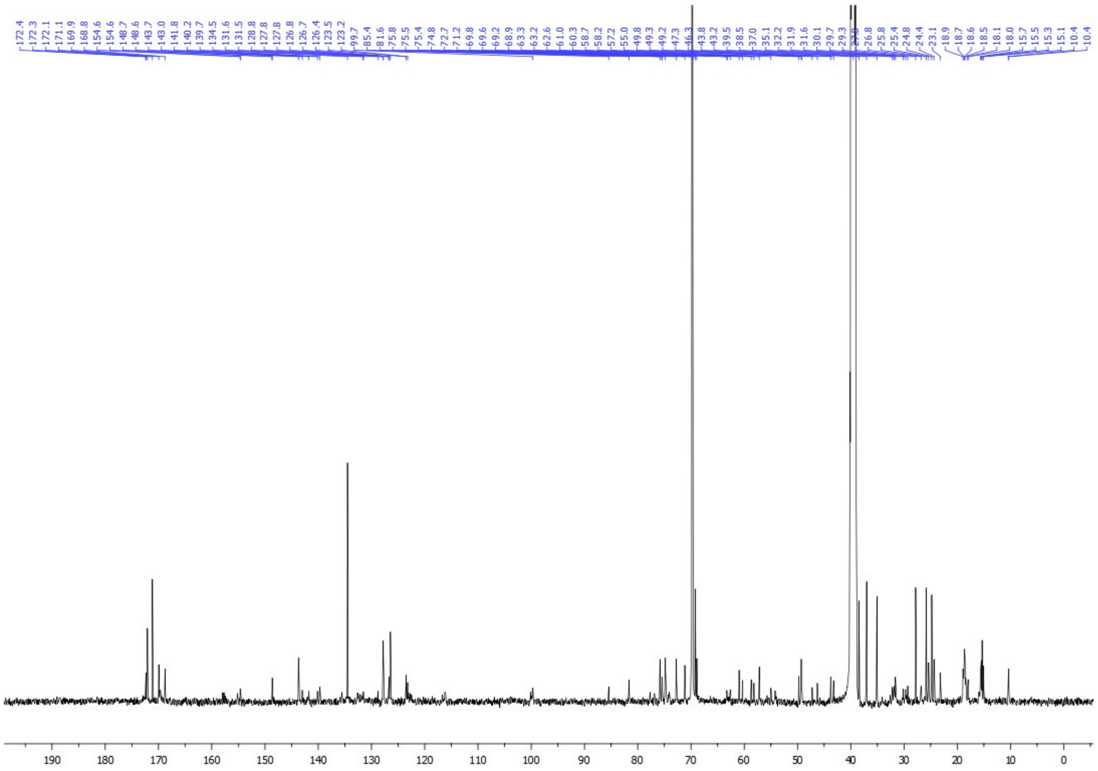

^{13}C NMR spectrum (125 MHz. 298 K. DMSO-*d*6) of **1**

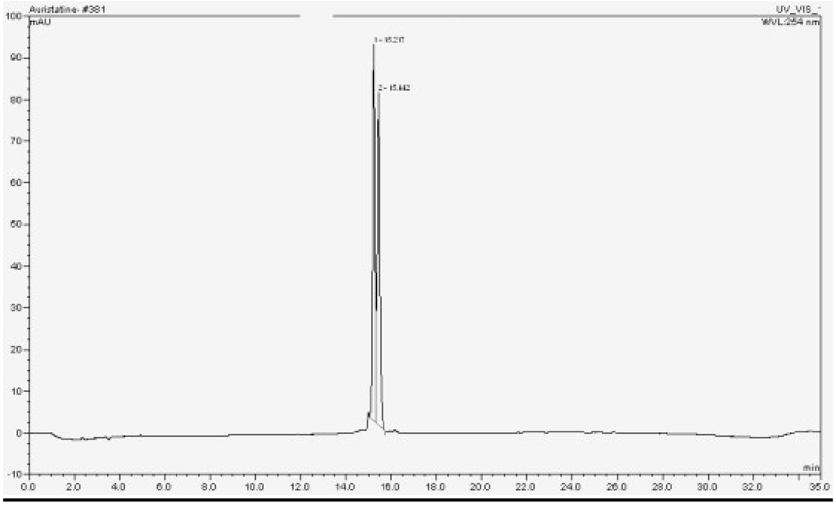

HPLC chromatogramm of **1** (as a mixture of two diastereoisomers**,** Method **1**) after semi-preparative reverse phase HPLC purification

II. Biology Section

II.1 Methods

Trypsin-digestion experiments: Prodrug **1** (0.33 $mg.mL^{-1}$) was incubated at 37°C in Human Serum Albumin (HSA) 5 % solution (purchased from Octapharma). After 3 hours under these conditions, the mixture was diluted 1:3 using an aqueous solution of 50 mM of ammonium bicarbonate and 10 mM of *tris*-(hydroxymetyl)aminomethane (pH 8.5). The resulting solution was successively treated with 10 µL of a 15.4 $mg.mL^{-1}$ DL-dithiothreitol solution at 60°C for 60 min and with 10 µL of a 44.4 $mg.mL^{-1}$ iodoacetamide solution at room temperature for 45 min in the dark. The mixture was then digested overnight using a 1:6 (w/w) trypsin-to-protein ratio. Digestion was stopped with formic acid at 0.4% final concentration and sample was analyzed by HPLC/HRMS/MS.

Enzymatic hydrolysis: Prodrug **1** (0.33 $mg.mL^{-1}$) was incubated at 37°C in Human Serum Albumin (HSA) 5 % solution (purchased from Octapharma) for 3 hours to form compound **2**. The mixture was diluted 1:3 using 0.02 M phosphate buffer (pH 7) and *Escherichia coli* β-glucuronidase (133 U/mL; purchased from Sigma Adrich) was added at 37°C. The release of MMAE along the time was followed by HPLC/HRMS/MS (screened MMAE m/z: 718.5117 $[M+H]^+$ for the precursor ion; 443,29099 $[M+H]^+$ and 686,48523 $[M+H]^+$ for the fragment ions).

Cells: MDA-MB-231-luc (D3H2LN) human breast cancer cell line was obtained from Perkin Elmer. MIA PaCa2 human pancreatic cell line was obtained from the American Type Culture Collection and stably transfected to express luciferase gene by Trichet's team, INSERM Nantes, France. A549 human lung carcinoma and KB human oral epithelial cancer cell lines were obtained from the European Collection of Authenticated Cell Cultures (ECACC). KB cells were stably transfected to express luciferase gene as previously described. All cells were grown in RPMI 1640-GlutaMAX supplemented by 10 % foetal bovine serum and 1 % Penicillin/Streptomycin in a humidified incubator at 37 °C and 5 % CO_2.

Cell viability: The Cell Proliferation Kit II XTT was used to measure cell viability. $2x10^3$ MDA-MB-231-luc or A549 and $4x10^3$ KB-luc or MIA PaCa2-luc cells/well were seeded in a 96-well plate, in RPMI 1640-GlutaMAX supplemented by 10 % foetal bovine serum and 1 % Penicillin/Streptomycin. Twenty-four hours later, cells were exposed to MMAE or **1** in presence or absence of *Escherichia coli* β-glucuronidase (40u/well). After 3 days of treatment, 25 µl of the XTT labelling mixture were added per well. After additional 4 h of incubation, absorbance was determined at 490 nm on a Berthold Mithras 96-well microplate reader. Experiments were performed 3 times in triplicate. Data were analysed with GraphPad software. For each compound, inhibitory concentration values (IC_{50}) were determined by the software for A549, KB-luc, MIA PaCa2-luc and MDA-MB-231-luc cells.

Experimental *in vivo* procedures: Female, 6 to 8 week-old Nude mice were purchased from Charles River Laboratories. Mice were acclimated for 7 days in the laboratory before experimentation and were maintained in sterilized filter-stopped cages inside a controlled ventilated rack and had access to food and water *ad libitum*. All experimental procedures involving animals (n°1031 for MDA-MB-231-luc, n°1065 for KB-luc and 1070 for MIA PaCa2-luc) were validated by the regional ethical committee (CECCO n°3) and carried out in accordance with the guidelines of the French Agriculture and Forestry Ministry (decree 2013-118) and of the European Communities Council Directive (2010/63/UE). All along the studies, mice were examined at least 3 times a week for clinical signs, distress, decreased physical activity and body weight as indicators of the health status.

Maximal Tolerated Dose studies (MTD): MTD studies were performed on BALB/c Nude mice (n = 3). A single administration of prodrug **1** (1 mg/kg, 2 mg/kg, 4 mg/kg, 8 mg/kg or 12 mg/kg) was performed by intravenous injection. Toxicity of **1** was evaluated by the maximum weight loss or gain, expressed as a percentage of the initial weight of the animals. A dose was considered as toxic if the relative weight loss was greater than 20 % of initial weight.

Bioluminescence imaging (BLI): BLI of mice was performed before the first treatment only to constitute homogenous groups of animals. Since this modality is strictly dependent upon ATP and oxygen, it cannot be used for assessment of proliferation in hypoxic tumours. So only ultrasound imaging was relevant for such a purpose. However, since BLI is the most sensitive modality to detect tumour cells *in vivo*, it was implemented at the end of studies to confirm the actual remission when tumour volumes became undetectable by echography. BLI images were obtained from an IVIS-Lumina II imaging system (Perkin Elmer) generating a pseudocoloured image representing light intensity and superimposed over a greyscale reference image. Each mouse was intraperitoneally injected with luciferin potassium salt at a dose of 100 mg/kg (purchased from Promega). Mice anesthetized by 1.5 % isoflurane were placed on a thermostatically controlled heating pad (37°C) during imaging. Acquisition binning and duration were set depending on tumour activity. Signal intensity was quantified as the total flux (photons/seconds) within ROIs drawn manually around the tumour area using Living Image 4.4 software (Perkin Elmer). For pancreatic tumours imaging, mice were placed on their right flanks. For breast tumours imaging, mice were placed in a supine position. For KB tumours imaging, mice were placed in a prone position. The thresholds of *in vivo* detection were better than 10^3 cells for breast tumours and around 2.10^4 cells for pancreatic tumours respectively.

Ultrasound imaging: Mice were anesthetized by inhalation of 1.5 % isoflurane with air and placed on a thermostatically controlled heating pad with the paws taped over the ECG electrodes attached to the table. Respiratory gating, derived from ECG, allows avoiding artefacts due to respiratory movements of the animal. Temperature of the animals was recorded with an internal temperature probe. An aqueous warmed ultrasonic gel (purchased from Supragel) was applied to the skin overlying the skin to optimize the visualization of internal organs. Tumours were imaged with the Vevo LAZR system (FUJIFILM Visualsonics Inc.). A transducer with central frequency at 40 MHz, providing axial resolution of 40 µm with a 14.1x15 mm field of view, was used for imaging of smaller tumours. A transducer with central frequency at 21 MHz, providing axial resolution of 75 µm with a 23.1x36 mm field of view, was used for larger tumour imaging. 3D scans of ultrasound image were recorded digitally and reviewed. The tumour area in a coronal plane was measured by manually delineating margins using Vevo LAB1.7.2 software (FUJIFILM Visaulsonics Inc.). The software then calculated the corresponding volume from each coronal slice, the threshold of detection ranging from $0.5mm^3$ to $1.5mm^3$ depending upon the tumour location.

Quantification of MMAE in KB xenografts: The relative quantity of MMAE was determined in KB tumour xenografts from mice (n = 4) treated with vehicle, MMAE (0.5 mg/kg), compound **1** (2 mg/kg), compound **3** (1.9 mg/kg or compound **4** (1.18 mg/kg). Vehicle treated mice were used as controls. 40 mg of tumours were lysed using a micropestle in 0.5 mL of 0.3 M sodium acetate and centrifuged for 5 min at 1500 xg. The supernatant was transferred in 1 mL of cold ethanol and incubated 1 h at -20°C. After centrifugation at 17000 xg and 4°C for 20 min, 0.5 mL of acetonitrile-methanol (2:1/v:v) were added to the supernatant and incubated 1 h at -20°C. The sample was then centrifuged for 5 min at 3000 xg. The supernatant was transferred to 2 mL microcentrifuge tubes and centrifuged again at 17 000 ×g and 4 °C for 20 min. Supernatants were analyzed by HPLC/HRMS on an Accela HPLC system coupled to a hybrid high resolution mass spectrometer Q-Exactive. 1 mL of

sample was injected and desalted by the mean of dual trap columns at a flow rate of 0.5 $mL.min^{-1}$ for 2 min with water-0.1 % formic acid as the loading eluent. Trapped analytes were then back flushed onto the analytical column (Acclaim C18 column, 250 x 4.6 mm, 5 µm, 120 Å) at 30°C. They were separated using a linear gradient composed of A (0.1% formic acid in water) and B (0.1% formic acid in CH_3CN), starting from 20 % of B and reaching 100 % of B within 30 min. Target selected ion monitoring data dependent-MS/MS (t-SIM-ddMS/MS) (ESI^+) was used to quantify MMAE in every extract. Targeted MS parameters were optimized as following: resolution of 70000 for precursor ion and 17500 for product ions, AGC target of 10^5 (precursor ion) and 2.10^5 (product ions), max IT of 100 ms (precursor ion) and 50 ms (product ions), MSX count 1, and isolation window of 2.0 m/z. The normalized collision energy was set at 35 %. The precursor ion selected for MMAE identification was $[M+H]^+$ m/z 718.509. The product ions m/z 86.097, 134.096 and 154.123 were selected to confirm MMAE identification. Peaks integration and MS spectra acquisition were performed with Thermo Xcalibur™ Qualitative Browser. A mass tolerance of 10 ppm was applied for the extraction of target product ions. The relative area of MMAE peak was determined in the chromatogram of every extract.

***In vivo* efficacy on subcutaneous oral epithelial tumours:** Human oral cancer xenografts from the KB epithelial cancer cell line were established in BALB/c Nude mice by subcutaneous implantation (n = 6). Mice were anesthetized by inhalation of 1.5 % isoflurane with air. The inoculum ($1x10^6$ tumor cells in 150 µL of a 50:50 Matrigel/PBS mix) was injected in the dorsal flanks of animals. Mice received treatment 7 days after tumour inductions. Five groups were designed and received once a week intravenous injections of a 5% DMSO and 95 % PBS mix (vehicle group), 0.5 mg/kg of free MMAE, 2 mg/kg of compound **1,** 1.9 mg/kg of compound **8** or 1.18 mg/kg of compound **9** during 4 weeks. Tumour volumes were determined by ultrasound imaging twice a week during 7 weeks after tumour implantation.

***In vivo* efficacy on orthotopic models:** Considering the obvious ethical issues, evaluation of new therapies and the understanding of biological mechanisms must be obtained from animal models. These animal studies correspond to the stage of proof of concept for the molecules which could be tested in clinical phase I and II. A key step in this preclinical process is to use a suitable model taking into account the clinical reality. A lack of clinical reality leads to a significant lack of predictability and a risky extrapolation of the results obtained *in vivo* in humans. It is necessary, to evaluate the efficiency of anti-tumour therapies, to perform these studies on orthotopic models. Orthotopic models have the advantage of being more predictive of tumour development in humans. The tumours growing in the original primary tumour tissue, these models are much closer to clinical pathological situation. Given the complexity of the processes involved, including interactions with the tumour microenvironment, no reliable method of replacement is available.

In vivo efficacy on orthotopic breast tumours: Human breast cancer xenografts from the MDA-MB-231-luc breast cancer cell line were established in BALB/c Nude mice by orthotopic implantation. Mice were anaesthetised by inhalation of 1.5% isoflurane with air. The inoculum ($2x10^6$ tumour cells in 100 µL PBS) was injected in the mammary fat pads of animals. Mice received treatment 15 days after tumour implantations. Three groups were designed and received once a week intravenous injections of a 5% DMSO and 95% PBS mix (vehicle group), 0.5 mg/kg of free MMAE or 4 mg/kg of compound **1** during 5 weeks (n = 6). Tumour volumes were determined by ultrasound imaging three times a week during 5 weeks after treatment initiation then weekly during 3 additional weeks.

In vivo efficacy on orthotopic pancreatic tumours: Human pancreatic cancer xenografts from the pancreatic cancer cell line MIA PaCa2-luc were established in Swiss Nude mice by orthotopic implantation. Mice were anaesthetized by inhalation of 1.5% isoflurane with air. Abdomens of mice were prepared with a solution of povidone iodine (Betadine). A small transverse incision was made in the left lateral flank through the skin and peritoneum. The tip of pancreatic tail was gently grasped and pancreas/spleen were externalized in a lateral direction to be fully exposed. The needle was inserted into the tail of pancreas and positioned in the pancreatic head region. The inoculum ($2x10^6$ MIA PaCa2-luc cells in 10 µL of PBS) was slowly injected using a 27-gauge needle of a Hamilton syringe. The spleen was then returned to the appropriate position in abdomen, peritoneum closed with 7-0 sutures and skin closed with 4-0 sutures.

Treatment of pancreatic tumours: Mice received treatment 7 days after orthotopic tumour xenografts. Four groups were designed and received once a week intravenous injections of a 5% DMSO and 95% PBS mix (vehicle group), 0.5 mg/kg of free MMAE during 3 weeks or 4 mg/kg of compound **1** during 2 weeks (n = 6). Tumour volumes were determined by ultrasound imaging three times a week during 3 weeks after treatment initiation then weekly during 7 additional weeks.

Treatment of pancreatic tumours with 2.5-3.5 cm³ initial volume: Mice received treatment 14 weeks after tumour xenografts. Intravenous injections were performed once a week during 9 weeks with a 4 mg/kg dose of compound **1** (n = 5). Tumour volumes were determined by ultrasound imaging weekly during 12 weeks after treatment initiation.

II.2 Characterization of compound 2

Trypsin digestion followed by HPLC/HRMS/MS analysis confirmed the formation of the coupling product **2** with the detection of the HSA peptide including the cysteine 34 linked to **1** (m/z 1070.5620 $[M+4H]^{4+}$, Fig. S1a). In the absence of **1**, the cysteine 34 is covalently bound to an acetamide group which comes from the iodoacetamide treatment (m/z 830.7668 $[M+3H]^{3+}$ Fig. S1b).

In the presence of β-glucuronidase, prodrug **2** led to the full release of MMAE (m/z 718.5117 $[M+H]^{+}$), in 50 min indicating that the glucuronide was readily substrate for the activating enzyme even with the proximity of the bulky albumin. Mechanism of drug release was confirmed by the detection of the HSA peptide fragment bound to residue resulting from the β-glucuronidase-mediated decomposition of the linker (m/z 1114.5730 $[M+3H]^{3+}$ Fig. S1c).

a)

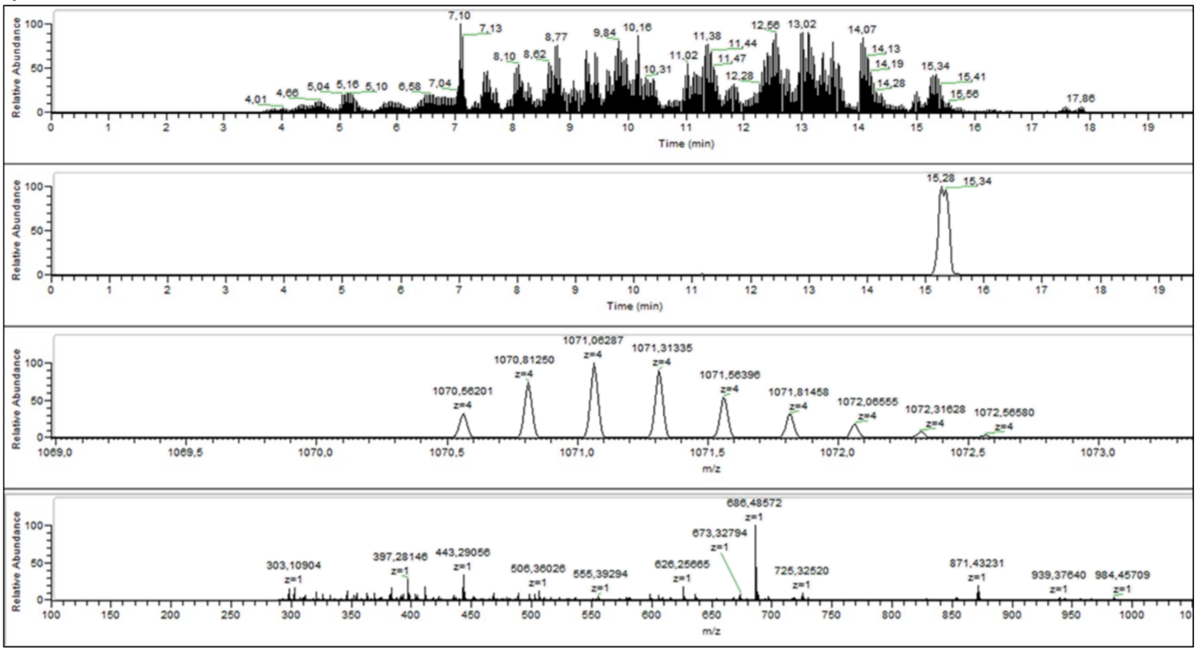

b)

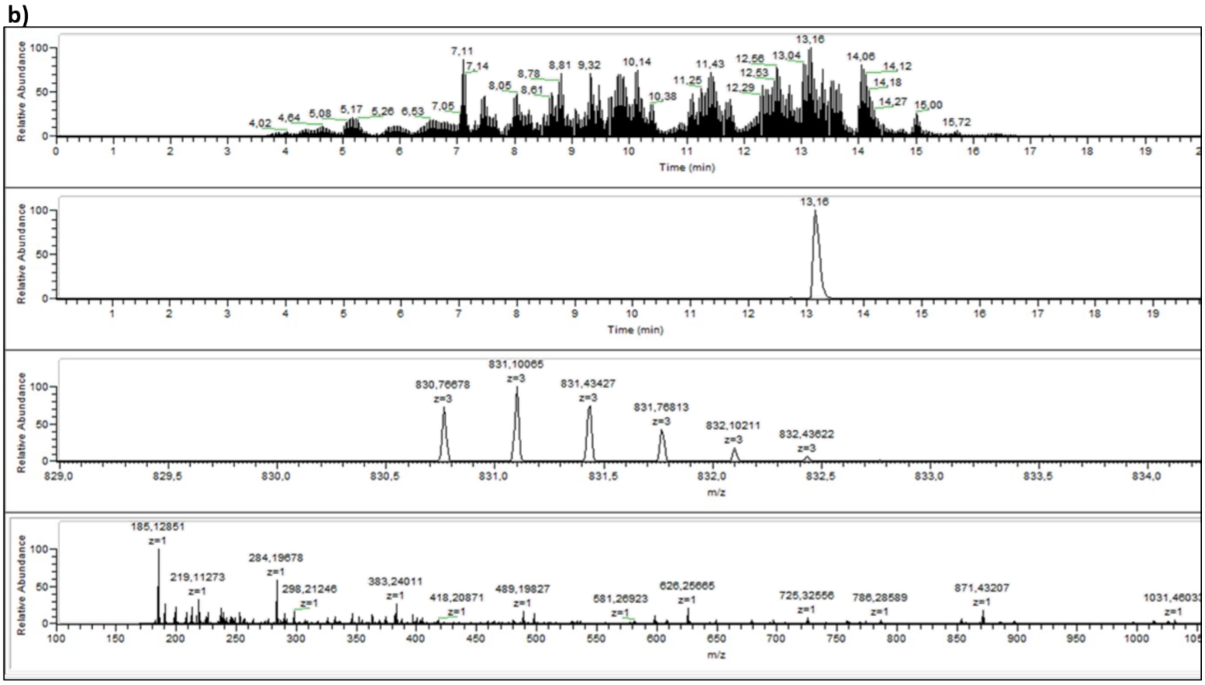

c)

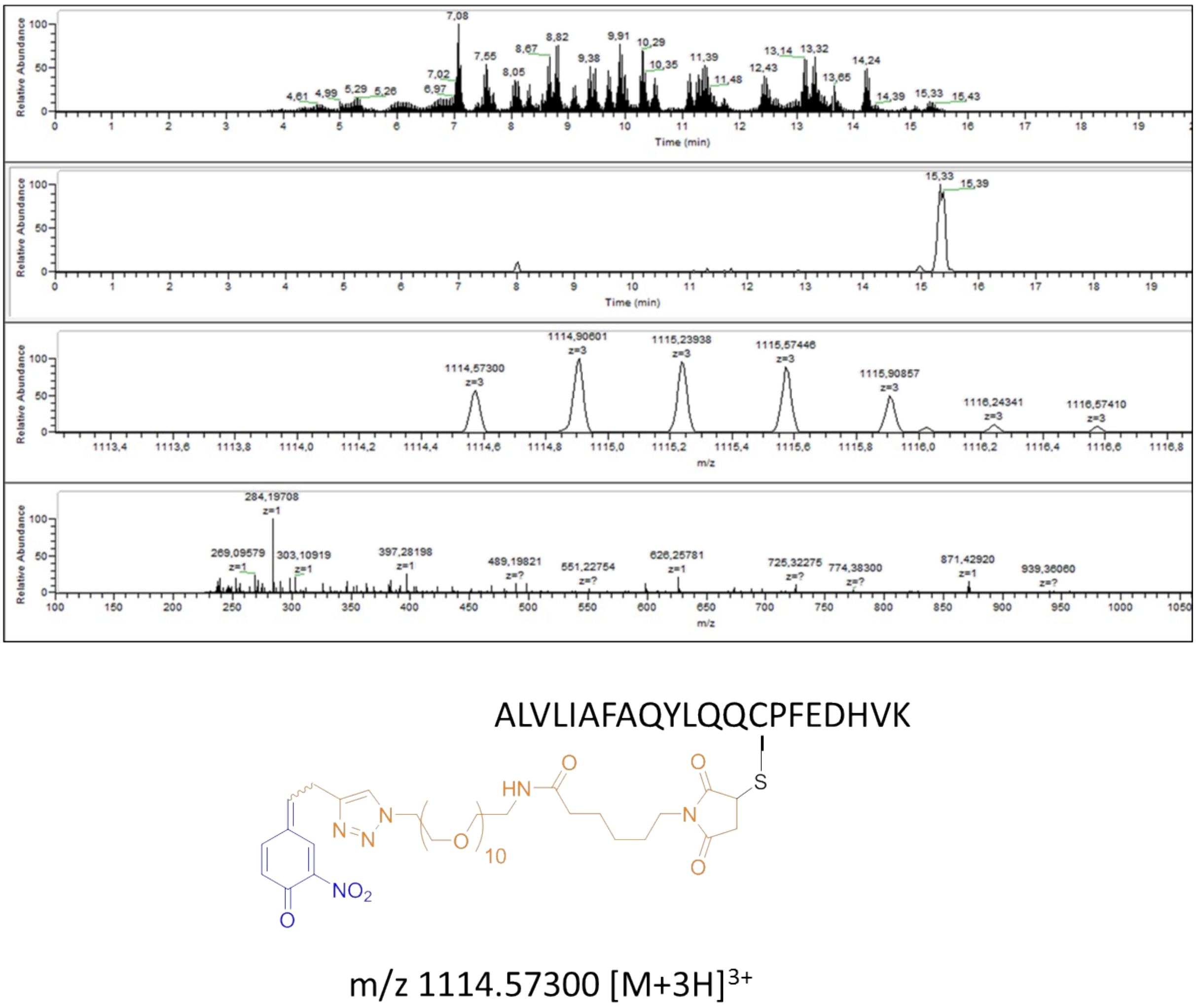

Figure S1. HPLC-HRMS/MS of trypsin digest of HSA incubated with or without 1. For each figure, from the top to the bottom: total ion chromatogram, chromatographic peak of a selected precursor ion, MS spectrum and finally MS/MS spectrum of this precursor ion. a) digest of **2**; b) digest of HSA; c) digest of **2** after β-glucuronidase hydrolysis.

II.3. IC50 values of prodrug 1

Cell line[a]	IC$_{50}$ values (nM) MMAE	1	1 + β-Glu
A549	2.48±0.57	208.46±94.54	4.65±1.64
KB	0.39±0.06	35.97±8.91	0.46±0.07
MIA PaCa2	0.73±0.09	76.20±11.79	0.84±0.14
MDA-MB-231	1.38±0.72	126.46±57.34	0.99±0.32

[a] A549 cells: human bronchial carcinoma; KB cells: human mouth epidermal carcinoma; MIA PaCa2 cells: human pancreatic carcinoma; MDA-MB-231 cells: human breast adenocarcinoma.

Table S1. IC$_{50}$ values (nM) of MMAE and prodrug **1** with or without β-glucuronidase (β-Glu) on A549, KB, MIA PaCa2 and MDA-MB-231 cell lines after 3 days of treatment. Values represent the mean ± SEM of three experiments performed in triplicate.

II.4. Tolerability studies for prodrug 1 and MMAE

To confirm the reduced toxicity of our prodrug *in vivo*, we conducted a tolerability study in tumour free Balb/c mice that received a single i.v. injection of **1** at doses ranging from 1 to 12 mg/kg. The body weights as well as clinical signs of toxicity were monitored regularly for fifteen days (Fig. S2). These assays demonstrated that glucuronide **1** was well tolerated up to doses of 8 mg/kg (contains 3.1 mg/kg of MMAE). In contrast, a 0.75 mg/kg dose of MMAE was highly toxic inducing a high rate of death in the animals, consistently with the previous data reported in the literature (Fig. S3). Therefore, the derivatisation of MMAE in the form of prodrug **1** markedly lowered its toxicity allowing at least the 4-fold administration of the lethal dose for the free drug.

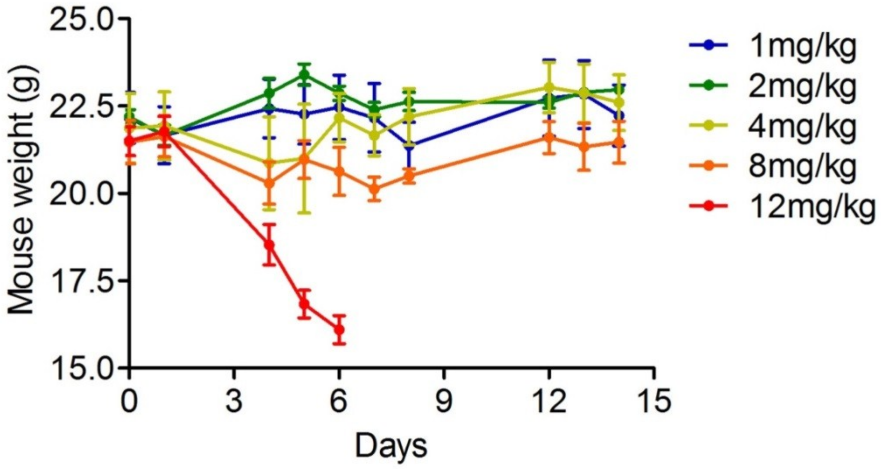

Figure S2. Mean body weights of mice treated with a single i.v. injection of **1** at 1, 2, 4, 8 or 12 mg/kg (day 0). Each point shows mean ± s.e.m. from 3 mice.

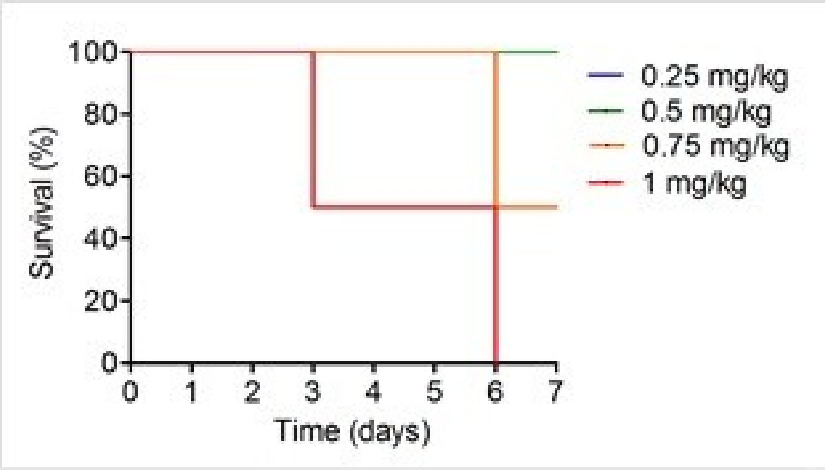

Figure S3. Survival curves of mice treated with free MMAE at 0.25 mg/kg, 0.5 mg/kg, 0.75 mg/kg or 1 mg/kg (two mice per group).

II.5. Histopathology analysis

Histological sections, cut at approximately 4 µm in thickness, were mounted on glass slides and stained with hemalun-eosin by Novaxia (Saint-Laurent Nouan, France) before submission to the pathologist, Le Net Pathology Consulting (Amboise, France). The quality of the histological sections, tissue accountability, slides labeling and tissue placement were considered adequate for the purposes of the study. The pathologist examined the tissue sections by light microscopy
on a Leica Diaplan microscope without knowledge of the treatment received by the mice. All histopathological findings on the organs were graded in severity using a five point system of minimal, slight, moderate, marked or severe.
Three animals were analyzed for each treatment. No abnormalities were found on duodenums, kidneys or livers. In summary, the administration of tested compounds in mice implanted with subcutaneous KB-neoplastic cells did not induce any compound-related effects.

II.6. Hypoxia in the Mia-PaCa2 orthotopic pancreatic tumour model

Immunostaining was performed with the Hypoxyprobe kit (purchased from hypoxyprobe), following the supplier's instructions. Mice received IV injection of 120 mg/kg of the pimonidazole solution. Animals were sacrificed 60 minutes following IV injection. Tumours were then resected and fixed for 48 h in buffered forlmaldehyde (10%) and placed in ethanol (70%). Tumours embedded in paraffin were sectioned with a 5µm thickness. A mouse anti-pimonidazole antibody (purchased from Abcam) was used as a primary antibody in order to label pimonidazole adducts as the hypoxia marker on the cross section of tumours. A goat FITC-labelled anti-mouse antibody (purchased from Abcam) was then used as a secondary antibody to highlight the presence of pimonidazole adducts. A DAPI mounting medium was used for staining cell nuclei in blue.

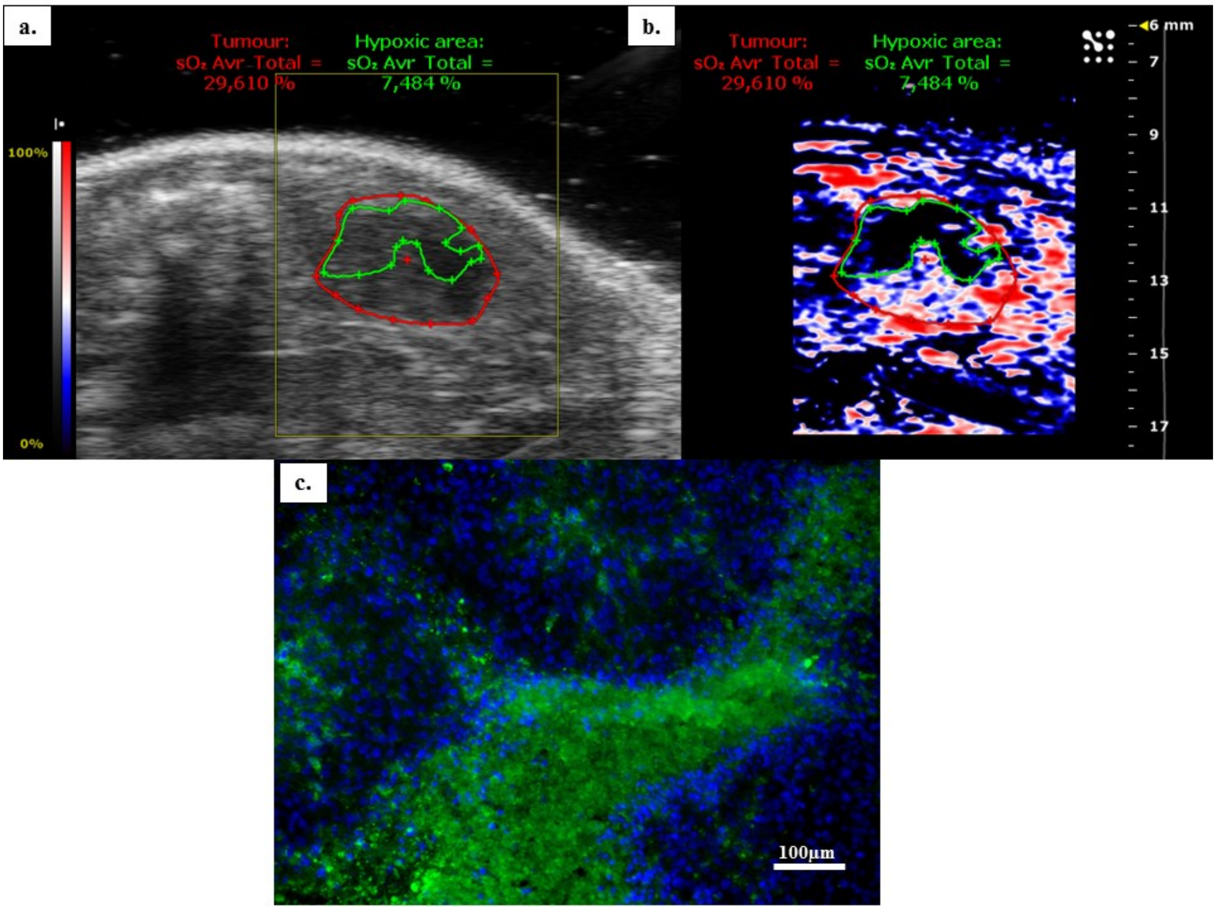

Fig. S4. Highlighting tumour hypoxia in the Mia PaCa2 orthotopic pancreatic tumour model. a & b, These illustrations present ultrasound B-Mode and corresponding OxyHemo photoacoustic images. These images are superimposed to determine the precise location of the hypoxic areas, thanks to the high resolution of the transducer. Blue and black areas correspond to low haemoglobin saturation with oxygen (SO_2), whereas red areas indicate where the saturation of haemoglobin is strong. In this 2D example, the saturation level of the haemoglobin is 29.6 % for the whole tumour area while it is about 7.5 % for the hypoxic area. **c**, Fluorescence images of a Mia-PaCa2 Anti-pimonidazole immunohistochemistry for hypoxia imaging. Pimonidazole adducts are labelled in green (FITC), and are concentrated as a core located in the photoacoustic low SO_2 area.

II.7. Pharmacokinetic study

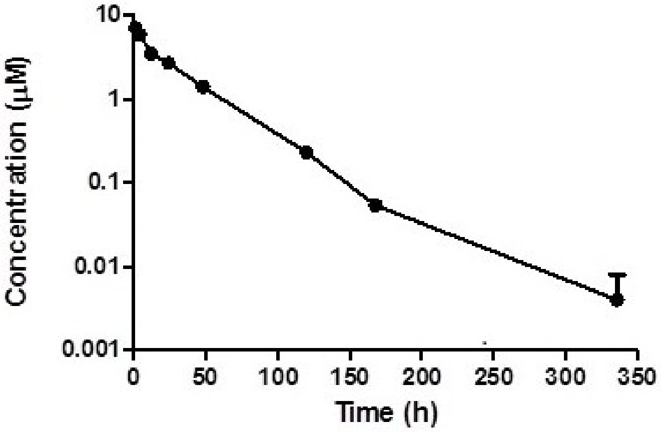

Fig. S5. Pharmacokinetics of prodrug 1 in mice.

Prodrug **1** was injected at 4 mg/kg in CD-1 mice (3 per time point) *via* the tail vein. At each time point (1h, 4h, 12h, 24h, 2d, 5d, 7d, 14d) a group of mice (n=3) was sacrificed, plasma samples were collected, freezed and stored at -80°C. Following the last plasma collection, all samples were defreezed. 200 μL of each sample were mixed with 400 μL of PBS and 60 μL of β-glucuronidase (100 U/mL) and incubated at 37°C in order to induce the release of the albumin-bound MMAE. After 14h of incubation, 1320 μL of MeCN were added in order to precipitate the proteins. Samples were vortexed for 3 minutes and then centrifuged (15000 g, 5 min at 4°C). Supernatants were analysed by LC-MS/MS and concentration of MMAE was determined by comparing MS signals with the standard calibration curve. A biological half-life of 35h was calculated assuming the first order kinetics of the elimination of the prodrug **1**.
